# mTOR activity and metabolic reprogramming of CD8^+^ T cells is impaired under hypoxia and within the multiple myeloma bone marrow

**DOI:** 10.1182/bloodadvances.2025016439

**Published:** 2025-09-15

**Authors:** Taylor Fulton-Ward, Nancy Gudgeon, Isaac Thirlwell, Emma L. Bishop, Bryan Marzullo, Hannah Giles, Graham McIlroy, Paul Ferguson, Bhuvan Kishore, Kate Rogers, Nuri Alfasi, Timothy Wong, Satnam Aytain, Daniel A. Tennant, Guy Pratt, Sarah Dimeloe

**Affiliations:** 1 Department of Immunology and Immunotherapy, School of Immunity, Infection and Immunology, College of Medicine and Health, University of Birmingham, Birmingham, United Kingdom; 2 Department of Metabolism and Systems Science, School of Medical Sciences, College of Medicine and Health, University of Birmingham, Birmingham, United Kingdom; 3 Centre for Clinical Haematology, University Hospitals Birmingham NHS Trust, Birmingham, United Kingdom

## Abstract

Novel therapies for multiple myeloma (MM) aim to engage antitumor functions of T cells. However, evidence indicates these functions are limited within the bone marrow (BM) environment. This is relatively hypoxic in health, and studies indicate widespread hypoxia in MM. In this study, CD8^+^ T-cell responses to stimulation were assessed under hypoxia, which identified that activation, proliferation, and interferon-gamma (IFN-γ) secretion were profoundly suppressed, while cytotoxicity and tumor necrosis factor-α (TNF-α) expression were unaffected. These changes occurred alongside decreased mechanistic target of rapamycin (mTOR) activity and expression of c-Myc, which drives T-cell metabolic reprogramming upon stimulation. Consistently, hypoxic CD8^+^ T cells demonstrated decreased activation-induced glycolysis and mitochondrial glutamine oxidation. Mechanistically, this was linked to elevated BCL2/adenovirus E1B 19 kDa protein-interacting protein 3 (BNIP3) expression under hypoxia, and reciprocally decreased abundance of its interaction partner, Ras homolog enriched in brain (Rheb), an important mTOR activator. Assessment of BCMA×CD3 bispecific antibody activity confirmed impaired capacity to elicit CD8^+^ T-cell activation, IFN-γ expression, proliferation, and altered memory differentiation under hypoxia, although initial target cell killing was unaffected. Finally, assessment of BM CD8^+^ T cells from patients with MM identified decreased proliferation, c-Myc, and Rheb expression compared with peripheral blood cells, alongside elevated BNIP3, confirming mechanistic features of hypoxic exposure in this environment. Taken together, the findings indicate potential for BM hypoxia to influence efficacy of T cell–directed therapies for MM.

## Key Points

• CD8^+^ T-cell activation, function, and metabolic reprogramming are suppressed by hypoxia, linked to decreased mTOR activity.
• Hypoxia impacts CD8^+^ T-cell activation by BCMA×CD3 bispecific antibody, and BM CD8^+^ T cells exhibit hypoxic features in myeloma.

## Visual Abstract

**Figure:**
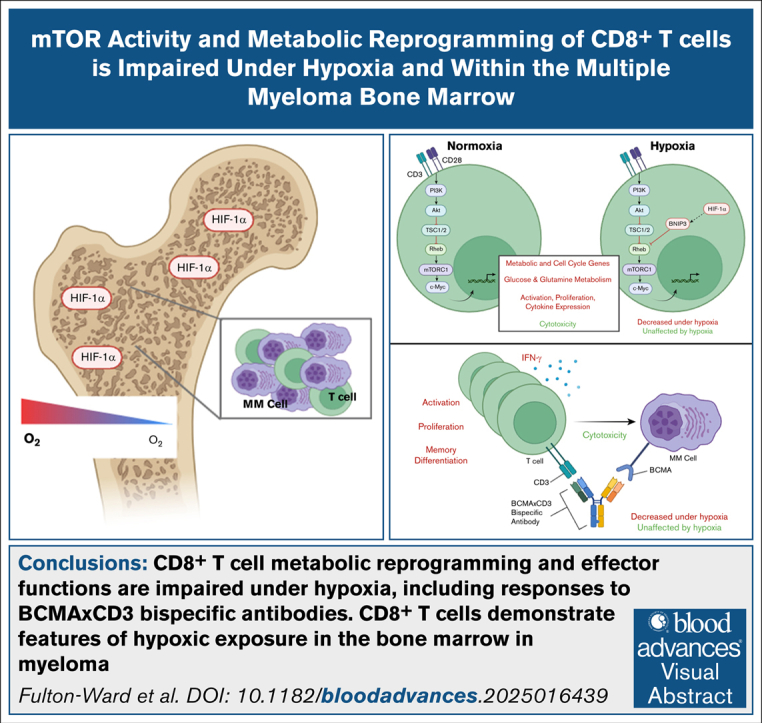

## Introduction

Multiple myeloma (MM) is a plasma cell malignancy that develops within the bone marrow (BM). Novel therapies, including bispecific antibodies, aim to exploit antitumor functions of T lymphocytes, including cytotoxicity and cytokine secretion. However, reports indicate these may be impaired within the BM in MM. For example, BM CD8^+^ T cells demonstrate reduced cytokine and cytotoxic molecule expression compared with those in peripheral blood of patients with MM,^1^ and T cells were found to be excluded from plasma cell-rich BM areas, with their activation status a gatekeeper of this.^2^

One factor that may impact BM T-cell function is oxygen availability. Physiological BM oxygen tensions are low (1.3%-5% O_2_),^3^^,^^4^ and further decrease in MM. Reports indicate abundant hypoxia-inducible factor 1-α (HIF1-α, which is stabilized below 3%-5% O_2_^5^, ^6^, ^7^) within MM BM trephines^8^, ^9^, ^10^ and in murine MM models.^11^^,^^12^ Elevated abundance of HIF1-α targets adrenomedullin and vascular endothelial growth factor (VEGF) within MM plasma cells, and VEGF and HLA-G in patient sera provide further evidence of BM hypoxia.^13^, ^14^, ^15^ Moreover, MM plasma cell proteomes exhibit features of hypoxic adaptation,^16^ and hypoxia-activated prodrugs demonstrate clinical activity, implying activation within the BM.^17^ Previous studies report hypoxia alters CD8^+^ T-cell activity, with both augmented and impaired activity described. These contradictory findings may relate to timing of exposure relative to T-cell activation. For example, studies consistently report that T cells previously activated and/or expanded under atmospheric oxygen tension (21% O_2_) increase activation marker, cytotoxic molecules, and cytokine expression upon subsequent hypoxic exposure.^18^, ^19^, ^20^, ^21^, ^22^, ^23^ However, when initial responses to T-cell stimulation are examined under hypoxia (typically 1% O_2_), studies generally agree that certain functions are suppressed, particularly proliferation and interferon gamma (IFN-γ) expression.^21^^,^^24^^,^^25^ Because T-cell responses to MM plasma cell antigens and bispecific antibodies are elicited within the hypoxic BM, the aim of this study was to profile how key effector functions of CD8^+^ T cells are impacted by exposure to hypoxia during activation. This has been modeled in peripheral CD8^+^ T cells from healthy donors stimulated via the T-cell receptor (TCR/CD3) and CD28 costimulatory receptor, to comprehensively profile functional and transcriptomic responses, and explore underpinning mechanisms. Relevance to T-cell–directed therapies in MM has also been probed by assessing bispecific antibody-elicited CD8^+^ T-cell activity and cytotoxicity, and analysis of BM and peripheral T-cell populations in patients with MM.

## Materials and methods

### Healthy volunteer peripheral blood donors

Peripheral blood mononuclear cells were isolated from fully anonymized leukocyte cones collected from NHS Blood and Transplant, Birmingham, United Kingdom. All volunteers provided consent, and studies were approved by the University of Birmingham STEM Ethics Committee (Ref. ERN 17_1743).

### MM BM aspirate and peripheral blood donors

BM aspirates and peripheral blood samples were obtained from patients with newly-diagnosed MM, recruited from University Hospitals Birmingham (supplemental Tables 1 and 2). The study received ethical approval (Ref: 10/H1206/58), and informed written consent was obtained.

### CD8^+^ T-cell isolation and culture

CD8^+^ T cells were isolated from peripheral blood by density-gradient centrifugation and positive selection (CD8 Microbeads, Miltenyi, catalog no. 135-045-201), purity was typically >95%. Cells were cultured at a density of 1 × 10^6^ cells per mL in RPMI-1640 containing 10% fetal calf serum, 50 U/mL penicillin, and 50 mg/mL streptomycin (Sigma, catalog no. P4333), and 50 IU/mL recombinant (r)IL-2 (PeproTech, catalog no. 200-02; RPMI/fetal calf serum) for 16 hours at atmospheric (21%) or reduced (5% or 1% O_2_; Don Whitley Hypoxystation) oxygen, to equilibrate oxygen tension prior to experiments. Cells were then activated using 12 μL/mL ImmunoCult Human CD3/CD28 T-Cell Activator (STEMCell, catalog no. 10971).

Analysis methods are provided in supplemental Material.

## Results

### CD8^+^ T-cell functional responses to stimulation via CD3/CD28 are limited under hypoxia

**Figure 1.**
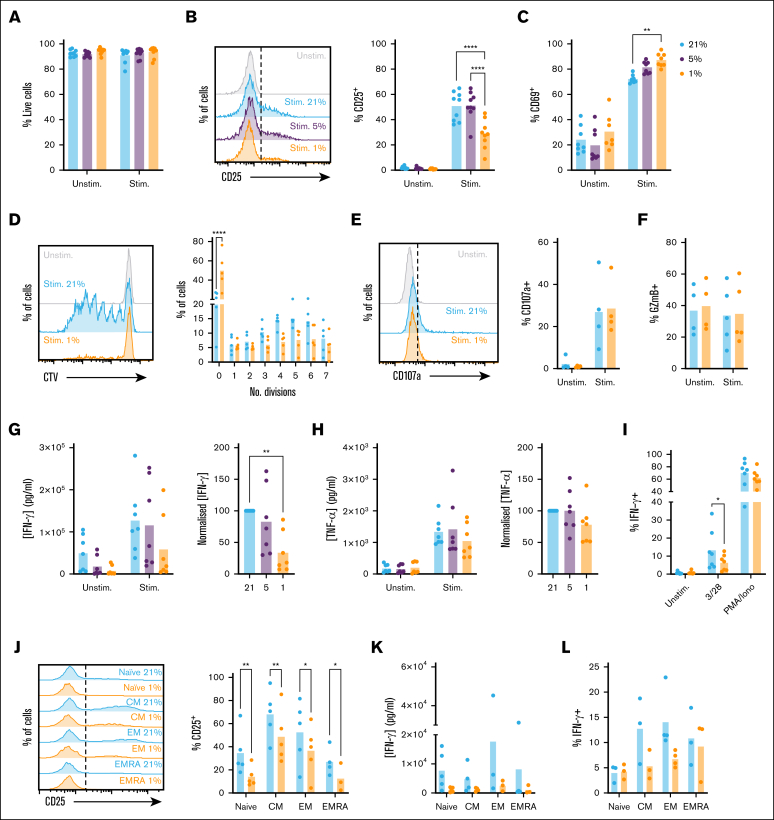
**CD8^+^ T-cell activation, proliferation, and IFN-γ expression are suppressed when stimulated under hypoxia.** (A-C) CD8^+^ T cells were cultured for 16 hours at 21%, 5%, or 1% O_2_, stimulated via CD3/CD28 as indicated at these oxygen tensions for 48 hours, then assessed for (A) viability, (B) CD25 expression, and (C) CD69 expression (representative histograms and summarized for n = 9 independent donors). (D) CD8^+^ T cells were cultured as in panel A, but stimulated for 6 days and assessed for proliferation by cell trace violet (CTV) dilution (representative histograms and summarized for n = 5 independent donors). (E-F) CD8^+^ T cells were cultured as in panel A, but stimulated for 5 hours in the presence of brefeldin A and monensin, and assessed for (E) CD107a trafficking and (F) intracellular granzyme B (GzmB) abundance (representative histograms and summarized for n = 4-5 independent donors). (G-H) Supernatants from CD8^+^ T cells cultured as in panel A were assessed for (G) IFN-γ and (H) TNF-α content (summarized for n = 7 independent donors, normalized to cytokine measured at 21% O_2_). (I) CD8^+^ T cells cultured as in panel A were restimulated at 48 hours via CD3/CD28 or with phorbol 12-myristate 13-acetate (PMA)/ionomycin as indicated, and assessed for intracellular IFN-γ abundance (summarized for n = 7 independent donors). (J-L) Sorted populations of naïve (CD45RA^+^CD62L^+^), CM (CD45RA^−^CD62L^+^), EM (CD45RA^−^CD62L^−^), and EMRA (CD45RA^+^CD62L^−^) cells were cultured as in panel A, and assessed for (J) CD25 expression, (K) IFN-γ secretion, and (L) intracellular IFN-γ abundance (summarized for n = 3-5 independent donors). *P* values were calculated by (B, C, D, J) 2-way analysis of variance (ANOVA) or (G) repeated measures ANOVA and Holm-Sidak’s post hoc test. ∗*P* < .05, ∗∗*P* < .01, ∗∗∗*P* < .001, ∗∗∗∗*P* < .0001.

To explore the impact of hypoxia on CD8^+^ T-cell function, we first assessed the activation status of cells stimulated via TCR/CD3 and CD28 receptors (CD3/28). Cells were rested at 1%, 5%, or 21% O_2_ for 16 hours to equilibrate oxygen tension prior to analysis. Selected oxygen tensions reflect the BM in MM, as evidenced by increased HIF-1α stabilization and activity^8^, ^9^, ^10^^,^^13^, ^14^, ^15^ (which occurs below 3%-5% O_2_^5^, ^6^, ^7^), physiological oxygen levels in the lymph node (5% O_2_), and atmospheric oxygen levels (21% O_2_), under which T-cell activity has typically been studied. Comparable viability was observed between conditions (supplemental Figure 1A; Figure 1A); however, cells cultured at 1% O_2_ demonstrated reduced upregulation of CD25 (interleukin-2 receptor high-affinity subunit) upon stimulation (Figure 1B; gating as in supplemental Figure 1A). Comparable CD25 was observed at 21% and 5% O_2_, indicating CD8^+^ T-cell activity is similar under these tensions, and that atmospheric oxygen (21% O_2_) can model oxygen levels within secondary lymphoid tissues. In contrast to CD25, CD69 was increased at 1% O_2_ (Figure 1C). CD69 is a sensitive marker of T-cell activation, but also a HIF-1α target, explaining this upregulation and limiting interpretation in hypoxia.^26^

To assess the impact of hypoxia on proliferation, CD8^+^ T cells were labeled with cell trace violet (CTV), equilibrated to 21% or 1% O_2_, activated and analyzed for CTV dilution after 6 days of activation. At this time point, CD8^+^ T cells demonstrated comparable viability (supplemental Figure 1B); however, proliferation was profoundly suppressed at 1% O_2_ (Figure 1D; supplemental Figure 1C; gating as in supplemental Figure 1A). To compare CD8^+^ T-cell cytotoxic potential under 21% and 1% O_2_, cells were stimulated in the presence of brefeldin A and monensin prior to intracellular staining for granzyme B and perforin A. Degranulation was monitored by inclusion of anti-CD107a antibody to label the membranal marker of degranulation as it cycled to the cell surface. This identified that CD8^+^ T-cell cytotoxic potential is not affected by hypoxia, with cells cultured at 21% and 1% O_2_ demonstrating similar CD107a trafficking (Figure 1E), and intracellular granzyme B and perforin A abundance. These were not increased upon activation, indicating they are largely preformed, which may explain insensitivity to hypoxia (Figure 1F; supplemental Figure 1D-E).

CD8^+^ T-cell expression of IFN-γ and tumor necrosis factor-α (TNF-α) was first assessed by enzyme-linked immunosorbent assay. This identified IFN-γ secretion was substantially lower from hypoxic cells (Figure 1G). Similar to CD25, IFN-γ secretion was comparable at 21% and 5% O_2_, while TNF-α secretion was comparable at all oxygen tensions (Figure 1H). TNF-α is rapidly secreted upon T-cell activation; lack of effect of hypoxia may thus relate to preformed cytokine. To test this, cytokine secretion was assessed from 8 to 72 hours of activation. Relatively little IFN-γ was secreted within the first 8 hours, but 50% of total secreted TNF-α was detected at this time point, confirming differential secretion kinetics (supplemental Figure 1F-G). Secreted IFN-γ reached 50% and 100% of total at 24 and 48 hours, respectively, and was decreased at all time points under hypoxia.

To probe the basis for impaired IFN-γ secretion, intracellular cytokine staining was performed. This identified that upon CD3/CD28 stimulation, intracellular abundance of IFN-γ was also decreased at 1% O_2_, indicating reduced secretion is partly explained by decreased protein expression (Figure 1I). Consistent with enzyme-linked immunosorbent assay data, intracellular TNF-α abundance was comparable in normoxic and hypoxic cells (supplemental Figure 1H). Of note, when CD8^+^ T cells were stimulated with phorbol 12-myristate 13-acetate and ionomycin, which bypass CD3/CD28 signaling pathways, intracellular IFN-γ abundance was comparable in CD8^+^ T cells cultured at 21% and 1% O_2_ (Figure 1I), indicating signaling defects impair IFN-γ expression in hypoxic cells.

### Naïve and memory CD8^+^ T-cell populations are equally affected by hypoxic exposure

CD8^+^ T cells comprise naïve (CD45RA^+^CD62L^+^), central memory (CM, CD45RA^–^CD62L^+^), effector memory (EM, CD45RA^–^CD62L^–^), and effector memory cells re-expressing CD45RA (EMRA) (CD45RA^+^CD62L^–^) subpopulations. Of these, EM and EMRA are most likely to encounter antigen within BM. However, these cells also generally demonstrate robust cytokine responses to antigen, supported by availability of preformed proteins and messenger RNA, and increased metabolic capacity, meaning they may better tolerate hypoxic environments. To probe this, naïve, CM, EM, and EMRA cells were purified by flow cytometry, and assessed for activation and cytokine expression. CM and EM subsets demonstrated increased capacity to upregulate CD25 upon activation vs naïve cells (supplemental Figure 1I; Figure 1J), while EMRA also demonstrated increased IFN-γ expression (Figure 1K-L). However, all groups were impaired in expression of both under hypoxia (Figure 1J-L), confirming findings in total CD8^+^ T cells apply to all subsets. TNF-α was not affected by hypoxia in any subset (supplemental Figure 1J).

### Activation-induced increases in CD8^+^ T-cell mTOR activity and metabolic reprogramming are limited under hypoxia

**Figure 2.**
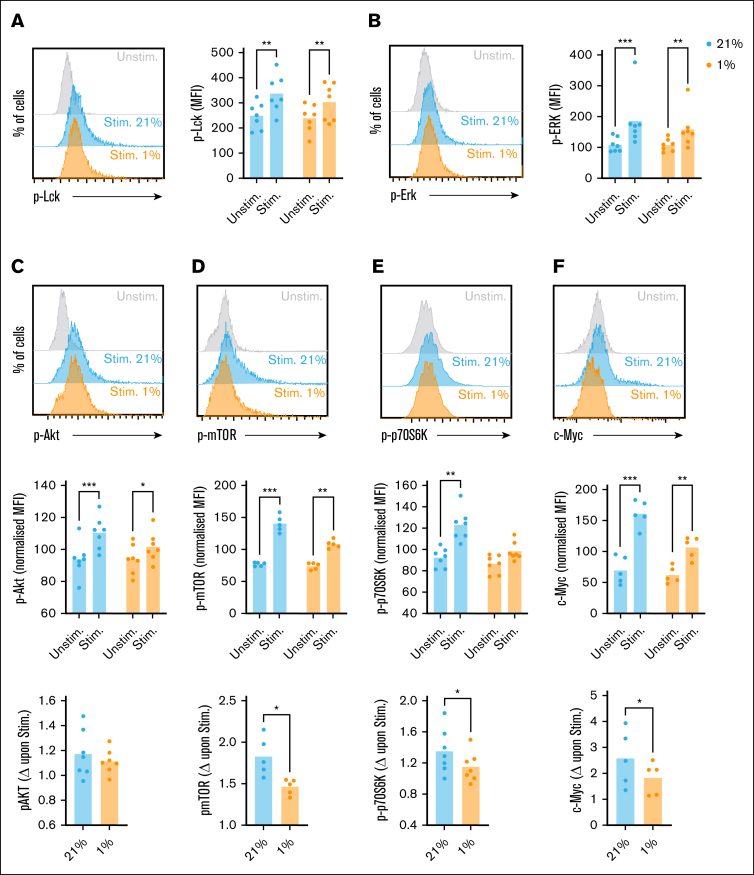
**CD8^+^ TCR signaling is intact when stimulated under hypoxia, but stimulation-induced mTOR activity and c-Myc upregulation are impaired.** CD8^+^ T cells were cultured for 16 hours at 21% or 1% O_2_, stimulated via CD3/CD28 at these oxygen tensions as indicated, as assessed for intracellular abundance of (A) phospho-Lck (p-Lck) after 15 minutes, (B) phospho-Erk (p-Erk) after 1 hour, (C) phospho-Akt (p-Akt), (D) phospho-mTOR (p-mTOR), and (E) phospho-p70S6 kinase (p-p70S6K) all after 24 hours, and (F) total c-Myc after 48 hours (summarized for n = 5-8 independent donors, shown as [A-B] mean fluorescence intensity [MFI], [C-F, upper] normalized to the average [mean] MFI for the 4 matched samples from each donor, or as stimulation-induced change [Δ], calculated as MFI of stimulated sample/MFI of matched unstimulated sample at each oxygen tension). *P* values were calculated by 2-way ANOVA and Holm-Sidak’s post hoc test or (lowest line) paired *t* test. ∗*P* < .05, ∗∗*P* < .01, ∗∗∗*P* < .001.

To understand if CD3/CD28 signaling in CD8^+^ T cells is altered under hypoxia, and define at which level this occurs, phospho-flow analysis of signaling proteins was performed. Within the TCR/CD3 signaling pathway, analysis of phospho-Lck (p-Lck) abundance identified expected increases in stimulated vs unstimulated cells, which were equivalent at 21% and 1% O_2_ (Figure 2A; gating as in supplemental Figure 1A). Similarly, abundance of phospho-Erk (p-Erk) was equally increased in normoxic and hypoxic CD8^+^ T cells upon stimulation (Figure 2B). CD28 enhances phosphoinositide 3-kinase activity to promote signaling via Akt and mechanistic target of rapamycin (mTOR). Analysis confirmed comparable increases in Akt phosphorylation (p-Akt) following stimulation of CD8^+^ T cells at 21% and 1% O_2_ (Figure 2C), but decreased abundance of phospho-mTOR (p-mTOR) and its target, phospho-p70-S6 kinase (p-p70S6K) in hypoxic cells (Figure 2D-E). Consistently, hypoxic CD8^+^ T cells demonstrated reduced abundance of the transcription factor c-Myc, translation of which is regulated by mTOR^27^ (Figure 2F). Measurement of total mTOR and p70S6K identified these were similar in normoxic and hypoxic CD8^+^ T cells (supplemental Figure 2A-B), confirming decreased phosphorylation rather than abundance of these proteins.

**Figure 3.**
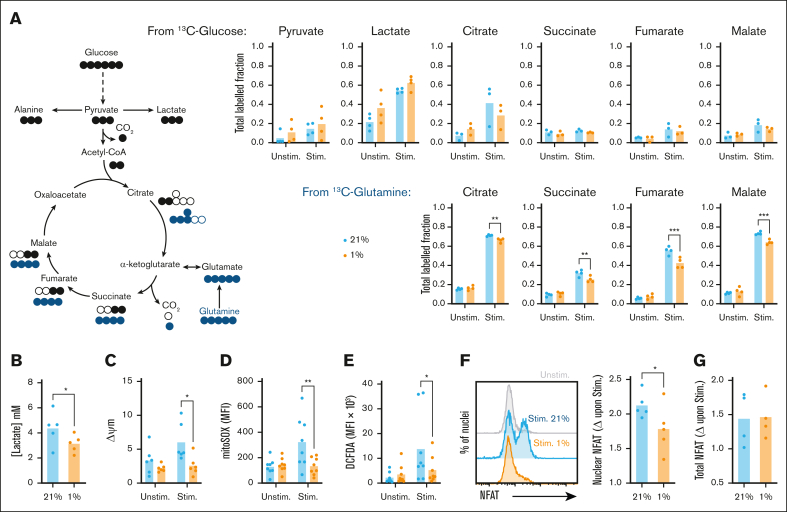
**Stimulation-induced increases in CD8^+^ T-cell metabolism, ROS production, and nuclear NFAT translocation are impaired under hypoxia.** (A) CD8^+^ T cells were cultured for 16 hours at 21% or 1% O_2_, stimulated via CD3/CD28 at these oxygen tensions as indicated for 24 hours in the presence of fully labeled ^13^C-glucose or ^13^C-glutamine, and assessed for fractional isotopic labeling of indicated metabolites by gas chromatography-mass spectrometry (GC-MS, summarized for n = 4 independent donors). (B) CD8^+^ T cells were cultured for 16 hours at 21% or 1% O_2_, stimulated via CD3/CD28 at these oxygen tensions as indicated for 48 hours, then assessed for excreted lactate by measurement of supernatant concentration (summarized for n = 5 independent donors). (C-G) CD8^+^ T cells cultured as in panel B were assessed after 24 hours for (C) mitochondrial membrane potential (Δψm, summarized for n = 6 independent donors), (D) mROS production (summarized for n = 8 independent donors), (E) total cellular ROS abundance (summarized for n = 8 independent donors), (F) nuclear (summarized for n = 5 independent donors), and (G) total NFAT abundance (summarized for n = 4 independent donors) within isolated nuclei and whole cells, respectively, all by flow cytometry. (F-G) Data are shown as change (Δ) calculated as MFI of stimulated sample/MFI of matched unstimulated sample. *P* values were calculated by (A-E) 2-way ANOVA and Holm-Sidak’s post hoc test or (F) paired *t* test. ∗*P* < .05, ∗∗*P* < .01.

Signaling via phosphoinositide 3-kinase-Akt-mTOR and upregulation of c-Myc support metabolic reprogramming of activated CD8^+^ T cells, promoting effector functions. To assess the impact of hypoxia on this, stable isotope-based tracing experiments interrogated metabolism of glucose and glutamine. CD8^+^ T cells were equilibrated to 21% and 1% O_2_, then activated for 24 hours with fully labeled ^13^C_6_-glucose or ^13^C_5_-glutamine (10 and 2 mM, respectively). Analysis of ^13^C incorporation from glucose identified increased labeling of pyruvate and lactate in stimulated vs unstimulated cells (Figure 3A; supplemental Figure 3A-E), consistent with increased glycolysis, but relatively little labeling into tricarboxylic acid (TCA) cycle intermediates. Conversely, labeling of these from ^13^C-glutamine increased upon stimulation, in agreement with glutamine being the major TCA substrate in activated T cells.^28^^,^^29^ At 1% O_2_, increased tendency for ^13^C-labeling of lactate from glucose in unstimulated cells indicated hypoxia-induced adoption of glycolysis. However, this did not consistently occur in stimulated CD8^+^ T cells, in which hypoxic cells excreted less overall lactate into the supernatant despite similar glucose consumption (Figure 3B; supplemental Figure 3F), indicating decreased activation-induced glycolysis. Analysis of glucose transporter messenger RNA abundance highlighted increased Glut3 (SLC2A3) but not Glut1 (SLC2A1) expression in resting CD8^+^ T cells at 1% vs 21% O_2_, potentially supporting observed increases in glycolysis (supplemental Figure 3G-H). Of note, transcripts for both transporters increased upon activation of hypoxic cells, which was not observed under normoxia. The apparent inconsistency of this observation with overall similar glucose uptake (supplemental Figure 3F) and metabolism highlights that nutrient intake is likely determined not only by transporter expression but also flux into metabolic pathways.

Consistent with impaired metabolic reprogramming, hypoxic CD8^+^ T cells also demonstrated reduced ^13^C incorporation from glutamine into TCA intermediates. This likely reflects combined effects on activation-induced metabolic reprogramming and direct alteration of mitochondrial activity under hypoxia. Indeed, increased abundance of M+5 citrate and M+3 malate isotopomers at 1% O_2_ (supplemental Figure 3D-E) indicates reductive carboxylation of glutamine-derived a-ketoglutarate to citrate, via reverse activity of isocitrate dehydrogenase, a described adaptation to impaired mitochondrial electron transport chain (ETC) function.^30^^,^^31^ Consistent with decreased ETC activity, CD8^+^ T cells activated at 1% O_2_ also demonstrated decreased mitochondrial membrane potential (Figure 3C) and reduced mitochondrial reactive oxygen species (mROS) generation vs 21% O_2_ (Figure 3D). mROS are a major component of total cellular ROS abundance, particularly in activated T cells, and consistently this was also decreased at 1% O_2_ (Figure 3E). In addition, mROS stabilize the transcription factor nuclear factor of activated T cells (NFAT), promoting nuclear translocation to drive T-cell activation.^32^ Assessment of NFAT within isolated nuclei identified activation-induced increases to be lower within hypoxic vs normoxic CD8^+^ T cells (Figure 3F; supplemental Figure 3I), despite total cellular levels being comparable (Figure 3G). Therefore, decreased NFAT nuclear translocation may also contribute to impaired CD8^+^ T-cell function under hypoxia.

### Transcriptomic analysis identifies decreased cell cycle gene expression in hypoxic CD8^+^ T cells and upregulation of BNIP3

**Figure 4.**
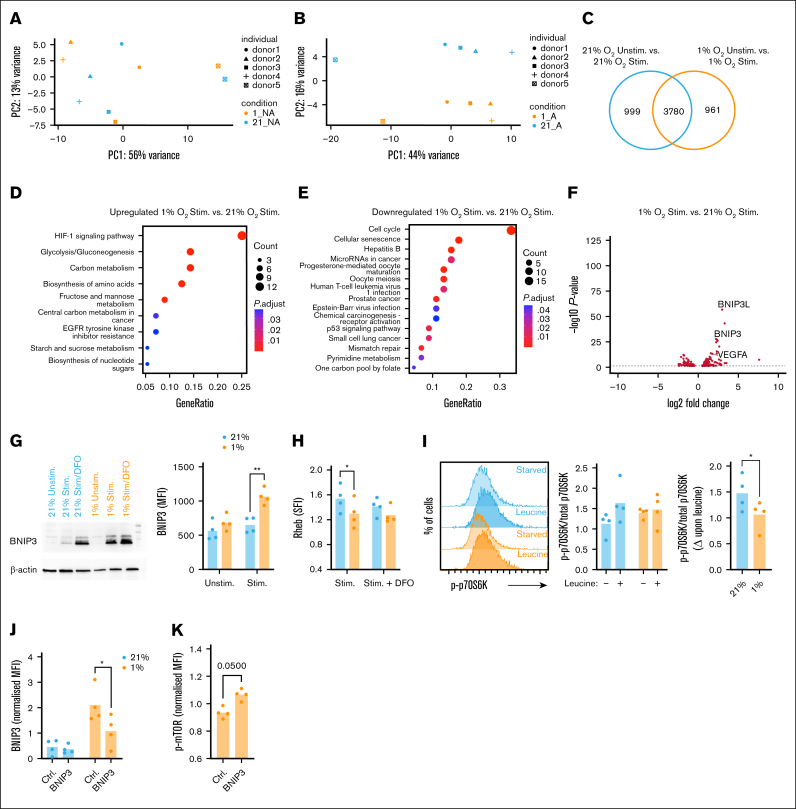
**Hypoxia alters stimulated CD8^+^ T-cell transcriptional profiles, inducing BNIP3 upregulation, decreased Rheb abundance, and impaired amino acid–induced mTOR activity.** CD8^+^ T cells were cultured for 16 hours at 21% or 1% O_2_, then left unstimulated or stimulated via CD3/CD28 for 24 hours at these oxygen tensions prior to RNA-sequencing analysis (n = 5 independent donors). (A-B) Principal component analyses identify unstimulated samples largely cluster by donor, whereas stimulated samples cluster by oxygen tension. (C) Venn diagram, with circles representing differentially expressed genes (DEGs) between indicated conditions to identify shared and distinct DEGs when comparing cells stimulated at 21% or 1% O_2_ with their respective matched unstimulated controls. (D-E) Pathway analysis of genes (D) upregulated or (E) downregulated in cells stimulated at 1% O_2_ compared with 21% O_2_. (F) Volcano plot of total transcript abundance in cells stimulated at 1% O_2_ compared with 21% O_2_. (G) Example western blot (left) and summary of intracellular flow cytometry analysis (right) of BNIP3 in CD8^+^ T cells stimulated as above for 24 hours, or additionally treated with the HIF-1α inducer, deferoxamine (DFO) where indicated (summarized data for n = 4 independent donors). (H) Summary of intracellular flow cytometry analysis of Rheb in CD8^+^ T cells stimulated as above for 24 hours (n = 4 independent donors). (I) CD8^+^ T cells were cultured for 16 hours at 21% or 1% O_2_, left unstimulated or stimulated via CD3/CD28 for 24 hours, serum starved for 6 hours then provided 10 mM leucine where indicated for 30 minutes, prior to measurement of intracellular phospho-p70S6 kinase (p-p70S6K) and total p70S6K by flow cytometry (representative flow cytometry histograms and summarized data for n = 4 independent donors, shown as ratio of p-p70S6K/total p70S6K and change (Δ) in this ratio upon leucine provision.) (J-K) CD8^+^ T cells were transfected with control (Ctrl.) or BNIP3-targeting CRISPR guide RNAs, and green fluorescent protein (GFP)-tagged Cas9, and stimulated at 21% or 1% O_2_ for 24 hours. (J) BNIP3 and (K) phospho-mTOR abundance within GFP^+^ cells, analyzed by flow cytometry (summarized data for n = 4 independent donors, normalized to the average [mean] MFI for matched samples from each donor). *P* values were calculated by (G, H, J) 2-way ANOVA and Holm-Sidak’s post hoc test, or (I, K) paired *t* test, ∗*P* < .05, ∗∗ *P* < .01.

To further explore how hypoxia impacts CD8^+^ T-cell activation and function, RNA-sequencing analysis was performed at rest after activation at 21% or 1% O_2_ for 24 hours. Principal component analysis indicated resting samples largely clustered by donor, but not oxygen tension (Figure 4A). Conversely, stimulated CD8^+^ T-cell samples separated by oxygen tension (Figure 4B), indicating hypoxia determines activation-induced CD8^+^ T-cell transcriptomes. Differentially expressed gene analysis identified ∼4000 genes commonly altered upon activation at 21% and 1% O_2_, and ∼1000 genes only altered in cells either activated at 21% or 1% O_2_, respectively (Figure 4C; supplemental Figure 4A-B). Pathway analysis revealed genes related to HIF-1α signaling, glycolysis/gluconeogenesis, and carbon metabolism were uniquely upregulated in cells activated at 1% O_2_ (Figure 4D), of which some were also upregulated in unstimulated hypoxic vs normoxic cells (supplemental Figure 4C). Those downregulated compared to cells activated at 21% O_2_ included genes related to cell cycle, in agreement with suppressed proliferation (Figure 4E). Specific genes highly upregulated in cells activated at 1% O_2_ included *VEGFA*, *BNIP3*, and *BNIP3L*, consistent with HIF-1α-dependent regulation (Figure 4F). Of these, BCL2/adenovirus E1B 19 kDa protein-interacting protein 3 (*BNIP3*) was of interest, as it mediates hypoxia-dependent inhibition of mTOR activity by interacting with Ras homolog enriched in brain (Rheb) to promote its degradation.^33^

### Hypoxic CD8+ T cells demonstrate decreased Rheb abundance and amino acid-dependent mTOR activation

Rheb is a guanosine triphosphate (GTP)-binding protein that localizes to the lysosomal membrane where it stimulates kinase activity of mTOR. This occurs through indirect and direct mechanisms, with the latter requiring the presence of extracellular amino acids, particularly leucine, thereby contributing to amino acid-dependent mTOR activation.^34^^,^^35^ Analysis at the protein level confirmed BNIP3 abundance was higher in CD8^+^ T cells activated at 1% compared with 21% O_2_, and that it was also increased by the HIF1-α–inducer deferoxamine (Figure 4G; supplemental Figure 4D-E). In agreement with BNIP3-dependent Rheb degradation, Rheb abundance was decreased in CD8^+^ T cells stimulated at 1% O_2_ and upon deferoxamine treatment (Figure 4H; gating as in supplemental Figure 1A). To directly probe whether amino acid-dependent mTOR activation was altered in hypoxic CD8^+^ T cells, cells stimulated at 21% and 1% O_2_ were serum-starved, then exposed to 10 mM leucine. Normoxic CD8^+^ T cells demonstrated a clear increase in mTOR activity upon leucine provision, as measured by phosphorylated vs total p70S6K (p-p70S6K/total p70S6K), but this did not increase in hypoxic cells, confirming impaired amino acid-induced mTOR activation (Figure 4I-J; gating as in supplemental Figure 1A). Measurement of regulated in DNA damage and development 1 (REDD1), which activates the mTOR suppressor tuberous sclerosis proteins 1/2 (TSC1/2) complex under hypoxia, revealed this to be comparable in hypoxic vs normoxic CD8^+^ T cells, particularly upon activation, indicating it may not influence mTOR activity in this context (supplemental Figure 4F). To directly probe whether BNIP3 regulates CD8^+^ T cell mTOR activity under hypoxia, BNIP3 expression was manipulated via CRISPR-Cas9 genome editing. CD8^+^ T cells were transfected with control or BNIP3-targeting guide RNAs together with green fluorescent protein-tagged Cas9, and stimulated at 21% or 1% O_2_. Flow cytometry analysis identified efficient transfection in all samples (supplemental Figure 4G), and that green fluorescent protein-positive cells treated with BNIP3-targeting guide RNA demonstrated reduced BNIP3 expression when stimulated under hypoxia compared with cells treated with control guide RNA (Figure 4J). Analysis of p-mTOR revealed this was consistently increased in BNIP3-targeted cells compared with controls under hypoxia (Figure 4K), which was not observed under normoxia (supplemental Figure 4H), confirming upstream regulation via BNIP3.

### BCMA×CD3 bispecific antibody-elicited CD8^+^ T-cell cytotoxicity of MM plasma cells is unaffected by hypoxia, but activation and cytokine secretion is suppressed

**Figure 5.**
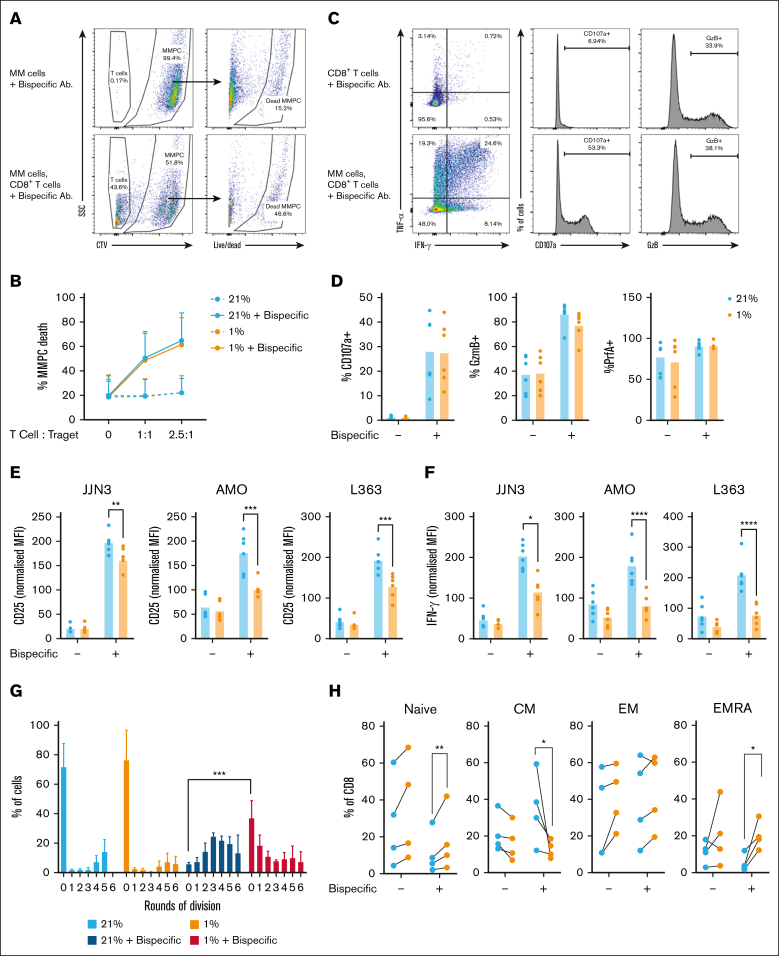
**Hypoxia does not affect initial CD8^+^ T-cell cytotoxicity elicited by BCMA×CD3 bispecific antibody, but limits CD25 and IFN-γ induction, proliferation, and alters memory differentiation.** (A-B) CD8^+^ T cells were cultured with BCMA-expressing, CTV-labeled JJN3 target cells and BCMA×CD3 bispecific antibody where indicated for 24 hours at either 21% O_2_ or 1% O_2_, prior to assessment of target cell viability (live/dead probe exclusion). (A) Representative flow cytometry plots, and (B) summarized data for n = 5 independent donors of CD8^+^ T cells across indicated T cell:target ratios. (C-D) CD8^+^ T cells and target JJN3 cells were cultured as above, additionally in the presence of brefeldin A/monensin and CD8^+^ T cells assessed for CD107a trafficking, intracellular granzyme B (GzmB), and perforin A (PrfA), by flow cytometry (C) representative flow cytometry plots/histograms, and (D) summarized data for n = 5 independent donors of CD8^+^ T cells at 2.5 T cells:1 target cell ratio. (E-F) CD8+ T cells were cultured as in panels C-D with indicated myeloma cell lines, and assessed for (E) surface CD25 and (F) intracellular IFN-γ by flow cytometry. Data are normalized to the average (mean) MFI for the 4 matched samples from each donor. (G-H) CD8^+^ T cells were activated as in panels A-B, and assessed after 7 days for (G) proliferation (carboxyfluorescein succinimidyl ester [CFSE] dilution), and (H) frequency of indicated populations as defined by surface expression of CD45RA and CD62L (summarized data for n = 4 independent donors of CD8^+^ T cells at 1 T cell:1 target cell ratio). *P* values were calculated by 2-way ANOVA and Holm-Sidak’s post hoc test. ∗*P* < .05, ∗∗*P* < .01, ∗∗∗*P* < .001, ∗∗∗∗*P* < .0001.

Novel therapies for MM engage antitumor T-cell function within the BM microenvironment. These include bispecific antibodies, which bind plasma cell targets, frequently B-cell maturation antigen (BCMA), together with T cell CD3, bringing the cells into proximity and eliciting T-cell cytotoxicity and cytokine production. To investigate the impact of hypoxia, an assay was established to measure bispecific antibody-induced plasma cell killing by CD8^+^ T cells, together with T-cell effector functions, and undertaken at 21% and 1% O_2_. CD8^+^ T cells were co-cultured with BCMA-expressing target myeloma cell lines and BCMA×CD3 bispecific antibody for 24 hours. Target MM cell lines were labeled with CTV dye to permit identification and quantification of cell death. Background levels of cell death were measured in cell lines cultured with BCMA×CD3 bispecific antibody without CD8^+^ T cells (Figure 5A-B). Parallel cultures were also established in which cells were treated with monensin and brefeldin A, permitting measurement of intracellular T-cell cytokines, granzyme B, and perforin A, alongside surface CD25 and CD107a trafficking, identifying T-cell functions elicited by BCMA×CD3 bispecific antibody. Certain CD8^+^ T-cell effector functions (ie, IFN-γ and TNF-α expression, CD107a trafficking) were only elicited when BCMA×CD3 bispecific antibody and target cells were present (Figure 5C), while intracellular granzyme B abundance was relatively high in unstimulated cells. Titratable increases in killing of 3 target MM cell lines were observed with increasing T cell:target ratios – JJN3 (Figure 5B), AMO, and L363 (supplemental Figure 5A-B). This only occurred in the presence of BCMA×CD3 bispecific antibody, and killing of all 3 cell lines was comparable at 21% and 1% O_2_ (Figure 5C; supplemental Figure 5A-B). In agreement, similar increases in CD107a trafficking were elicited by BCMA×CD3 bispecific antibody/target cells at both oxygen tensions, and intracellular abundance of granzyme B and perforin A was comparable (Figure 5D; supplemental Figure 5C-D).

In contrast, analysis of T-cell activation and cytokine expression (T cells gated as CTV^–^, as indicated in Figure 5A) identified markedly reduced capacity of BCMA×CD3 bispecific antibody/target cells to induce CD25 at 1% vs 21% O_2_ (Figure 5E-F). TNF-α induction also tended to be decreased at 1% O_2_, albeit more variably (supplemental Figure 5E). Elicited IFN-γ was, however, consistently and markedly reduced at 1% O_2_, with mean fluorescence intensity values (representing intracellular cytokine abundance) being on average 50% less than at 21% O_2._ Taken together, the findings indicate initial BCMA×CD3 bispecific antibody-elicited CD8^+^ T-cell cytotoxicity is unaffected by hypoxia, potentially explained by the presence of preformed cytotoxic mediators, but that T-cell activation and IFN-γ expression are suppressed. Because CD25 upregulation underpins T-cell expansion and differentiation, the impact of hypoxia on these responses to BCMA×CD3 bispecific antibody was also assessed after 7 days. Viability of CD8^+^ T cells was comparable at 21% and 1% O_2_ in both resting and stimulated cells (supplemental Figure 5F). However, T-cell proliferation upon BCMA×CD3 bispecific antibody/target cell stimulation was decreased at 1% vs 21% O_2_, as evidenced by a larger percentage of undivided cells (Figure 5G). In addition, T-cell differentiation after bispecific antibody treatment was altered, with increased frequency of naïve and EMRA cells at 1% O_2_ compared with 21% O_2_, and substantially fewer CM cells (Figure 5H).

### CD8^+^ T cells within the MM BM demonstrate decreased proliferation and function, alongside mechanistic features of hypoxic exposure

**Figure 6.**
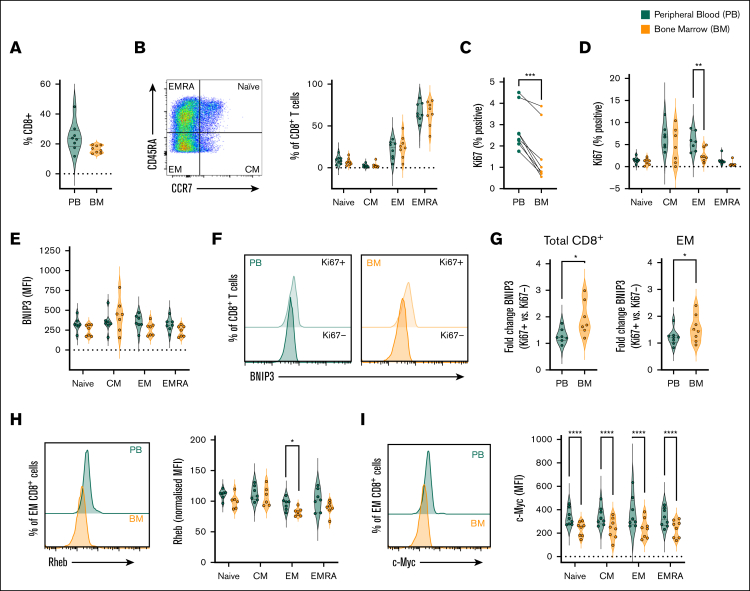
**BM CD8^+^ T cells exhibit impaired proliferation, elevated BNIP3, and decreased c-Myc and Rheb expression in MM.** Paired BM and peripheral blood (PB) mononuclear cells from patients with newly diagnosed MM were analyzed by flow cytometry for: (A) frequency of CD8^+^ T cells; (B) frequency of naïve, CM, EM, and EMRA populations within total CD8^+^ T cells; (C-D) frequency of Ki67-expressing cells within (C) total CD8^+^ T cells and (D) indicated populations, (E-G) BNIP3 expression within (E) total indicated populations and (F-G) Ki67^+^ and Ki67^−^ cells, (F) representative histograms of total CD8^+^ T cells, and (G) fold change of BNIP3 abundance in Ki67^+^ vs Ki67^−^ cells of indicated populations, (H) Rheb abundance (representative histograms of total CD8^+^ T cells and mean within indicated populations), and (I) c-Myc abundance (representative histograms of total CD8^+^ T cells and mean within indicated populations). For all analyses n = 8 independent paired samples, data are shown as (B-D) percentage of parent population, or (E-I) MFI. Where normalized (H), this is to the average (mean) MFI of all populations from matched samples for each individual patient. *P* values were calculated by (C,G) paired *t* test, and (D, H, I) 2-way ANOVA and Holm-Sidak’s post hoc test. ∗*P* < .05, ∗∗*P* < .01, ∗∗∗*P* < .001, ∗∗∗∗*P* < .0001.

Finally, to assess whether features of hypoxia-induced suppression of function and mTOR activity are observed in BM CD8^+^ T cells in MM, these were assessed and compared with matched peripheral blood cells from patients with newly-diagnosed disease. Overall, CD8^+^ T-cell frequencies were similar in peripheral blood and BM (gating strategy as supplemental Figure 6A; Figure 6A). These comprised large proportions of EM and EMRA cells (Figure 6B), consistent with cohort age (supplemental Tables 1 and 2). Analysis of antigen kiel 67 (Ki67) expression identified abundant Ki67^+^ proliferating cells within CM and EM populations, in agreement with heightened proliferative capacity, which were significantly reduced within all BM populations compared with peripheral blood (supplemental Figure 6B; Figure 6C-D; gating as in Figure 6B). Analysis of intracellular IFN-γ and TNF-α upon ex vivo CD3/CD28 stimulation identified memory and EMRA cells as the major producers of these cytokines (supplemental Figure 6C-D; gating as in Figure 6B). As previously reported,^1^ cytokine expression tended to be lower in BM cells vs peripheral blood, albeit this did not reach significance in the smaller cohort analyzed here.

BNIP3 abundance was similar between peripheral blood and BM CD8^+^ T cells when total populations were analyzed (Figure 6E). However, in agreement with expression being induced upon CD8^+^ T-cell stimulation (Figure 4), it was markedly increased in Ki67^+^ cells, which have entered cell cycle in response to antigenic stimulation (Figure 5F; gating as in supplemental Figure 6B), as also indicated by decreased expression of CD45RA, which is downregulated upon TCR stimulation (supplemental Figure 5E). Notably, fold increases of BNIP3 expression in Ki67^+^ vs Ki67^–^ cells was greater in BM CD8^+^ T cells than those from peripheral blood (Figure 5G), recapitulating effects of activation under hypoxia observed in vitro. Among CD8^+^ T-cell subsets, BNIP3 upregulation in BM cells was clearest in EM cells (Figure 5G; supplemental Figure 6F), although analysis of Ki67^+/−^ subpopulations of naïve and EMRA cells is limited by their paucity (supplemental Figure 5B). Consistent with increased BNIP3 in BM CD8^+^ T cells, Rheb abundance was reduced within BM populations, again particularly EM cells, indicating reduced capacity for mTOR activation (Figure 6H; gating as in Figure 6B). While it was not possible to reliably measure p-mTOR or p-p70S6K abundance within cryopreserved samples, analysis of the more stable c-Myc identified this was reduced in all BM CD8^+^ T-cell populations (Figure 6I; gating as in Figure 6B), again particularly within EM CD8^+^ T cells. Taken together, this paired analysis of BM and peripheral CD8^+^ T cells indicates reduced CD8^+^ T-cell proliferative and effector function within the BM environment in MM, alongside increased BNIP3 abundance in activated cells, and decreased levels of the mTOR activator Rheb and target c-Myc, thereby reflecting mechanistic features of T-cell suppression induced by hypoxic exposure.

## Discussion

Here, the impact of hypoxia on CD8^+^ T cells was assessed and probed for relevance in MM by examining T-cell–directed therapies and interrogating patient samples. Hypoxic exposure profoundly suppressed CD8^+^ T-cell proliferation and IFN-γ expression upon activation, but not cytotoxicity or TNF-α expression. Effects on CD4^+^ T cells were not explored, but include suppression of IFN-γ and promotion of immune-suppressive regulatory T cells.^36^^,^^37^ Conversely, HIF-1α promotes metabolism and cytokine production, suppressing regulatory T cells-associated FoxP3 expression, in previously-activated CD4^+^ T cells exposed to hypoxia, highlighting complex interactions between hypoxia and T-cell activation status, as for CD8^+^ T cells.^38^

Differential effects of hypoxia on expression of IFN-γ vs TNF-α and cytotoxic molecules may relate to differential synthesis requirements, as granzyme B and perforin A are present in unstimulated CD8^+^ T cells, which also rapidly release TNF-α upon stimulation, whereas IFN-γ requires longer, indicating a requirement for transcription and translation. Because these are metabolically demanding processes, it is consistent with decreased CD8^+^ T-cell metabolic reprogramming under hypoxia that IFN-γ expression is particularly sensitive. Moreover, IFN-γ expression is directly linked to T-cell metabolism, via glucose metabolism-dependent transcriptional and post-transcriptional regulation.^39^, ^40^, ^41^ The profound suppression in cell proliferation observed under hypoxia may also relate to reduced metabolic capacity, alongside altered expression of cell cycle pathway genes. Indeed, hypoxia suppresses cycling of diverse cell types via HIF-1α–dependent increases in cyclin inhibitors p27 and p21, cell division cycle associated 2 (CDCA2), and prolyl-hydroxylase-mediated effects on p53 stability.^42^

The findings indicate impaired CD8^+^ T-cell metabolism and function under hypoxia relate to decreased mTOR activity and c-Myc expression. Moreover, c-Myc is decreased within BM CD8^+^ T-cell populations from patients with MM, which also demonstrate impaired cytokine expression and proliferation. c-Myc is a key driver of T-cell metabolic activity, promoting glucose and amino acid transporter expression, alongside enzymes mediating glucose and glutamine metabolism.^43^^,^^44^ Consistently, c-Myc–deficient T cells demonstrate poor growth and proliferation. Impaired c-Myc expression is described in other scenarios of immune-metabolic dysfunction, including obesity.^45^^,^^46^ Because the BM is a lipid-rich environment, in which CD8^+^ T cells demonstrate elevated lipid uptake capacity,^1^ this may also contribute to decreased c-Myc in this context.

Hypoxia-induced decreases in mTOR activity are also described in T-cell acute lymphoblastic leukemia, which also develops within the BM. Similar to our observations, hypoxic exposure impaired proliferation and mitochondrial activity of these cells, associated with decreased mTOR activity, restored by HIF1-α knockdown.^47^ HIF1-α–dependent regulation of BNIP3 and Rheb may again contribute, although upregulation of REDD1, which activates the mTOR suppressor TSC1/2 complex, was also described. In the current study, REDD1 expression was not increased in hypoxic CD8^+^ T cells, indicating it likely does not influence mTOR activity of nonmalignant CD8^+^ T cells in the BM, but additional mechanisms of hypoxia-induced mTOR suppression, for example, AMP-activated protein kinase (AMPK)-dependent TSC1/2 activation or promyelocytic leukemia (PML)-mediated mTOR nuclear sequestration were not explored.^48^

Similar to observations under CD3/CD28 stimulation, BCMA×CD3-elicited cytotoxicity was comparable under normoxic and hypoxic conditions, consistent with equivalent cytolytic molecule expression and degranulation, indicating this key aspect of bispecific antibody activity may withstand BM hypoxia. Conversely, BCMA×CD3-elicited IFN-γ and CD25 expression were substantially limited under hypoxia, alongside proliferation. T-cell differentiation in response to BCMA×CD3 bispecific antibody was also altered, with increased naïve and EMRA cells observed, and fewer CM cells, considered critical for sustaining long-lived responses. Relative importance of distinct T-cell activities, expansion, and differentiation for effective therapeutic responses is yet to be fully defined. In murine MM models, bispecific antibodies promote rapid plasma cells clearance, indicating they initially engage nascent cytotoxicity of T cells in situ in the BM.^49^ However, durable therapeutic responses likely depend on sustained T-cell expansion, differentiation, and cytokine secretion. Indeed, CD8^+^ T-cell clonal expansion is reported in patients receiving bispecific antibody therapy for MM, with expansion of effector-like clones associated with therapeutic responsiveness.^50^

Taken together, our data identify hypoxic exposure during CD8^+^ T-cell stimulation substantially impacts their transcriptional, metabolic, and functional status. CD8^+^ T cells within the BM in MM demonstrate features of these hypoxia-induced metabolic and functional changes. This may impact efficacy of T-cell–directed therapies in MM, therefore targeting these features could be explored as orthogonal therapeutic approaches. Examples of such therapies include hypoxia-activated prodrugs, oxygen-generating nanocarriers, or compounds that directly target HIF1-α synthesis or dimerization.^51^^,^^52^ Of note, combined immune-directed therapy (checkpoint blockade) and hypoxia-activated prodrugs demonstrate promising synergy in prostate cancer.^53^ One such drug, TH-302, was also effective in preclinical myeloma models, progressing to a clinical trial, albeit not in combination with bispecific antibody. Direct targeting of HIF-1α has also been reported to reduce disease burden in murine myeloma models,^54^^,^^55^ although immune cell activity was not assessed here.

Conflict-of-interest disclosure: D.A.T. undertakes paid consultancy work for Sitryx Ltd. The remaining authors declare no competing financial interests.
