# Supplementary material for: mTOR activity and metabolic reprogramming of CD8+ T cells is impaired under hypoxia and within the multiple myeloma bone marrow
Source: Blood Adv. 2025 Sep 15;9(23):6175–88. doi: 10.1182/bloodadvances.2025016439 (PMC12719155; doi:10.1182/bloodadvances.2025016439)
Supplement: Supplemental Methods, Tables, and Figures [file BLOODA_ADV-2025-016439-mmc1.pdf]

Supplemental Material

Supplemental Materials and Methods

Table S1

Table S2

Table S3

Supplementary Figures 1-6

Supplemental Materials and Methods

Flow cytometry cell sorting

CD8$^+$ T cells were isolated from human PBMC as above before surface staining with anti-human CD45RA, CD62L, CD8 antibodies (**Table S3**) in sterile FACS buffer for 20 minutes on ice. Cells were washed with FACS buffer before resuspension in SILAC™-RPMI 1640 Flex Media (Gibco, catalogue #A24942-01) with 1% FCS, penicillin/streptomycin and 100 IU/ml IL-2 at a concentration of 30 x 10^6/ml. Cells were filtered in a 75 µm mesh FACS tube prior to sorting. Naïve, effector memory (EM), central memory (CM), and terminally differentiated effector memory cells re-expressing CD45RA (EMRA) CD8+ T cells were collected into separate sterile FACS tubes containing RPMI-1640 media with penicillin and streptomycin, 20% FCS and 100IU/ml IL-2 and subsequently resuspended as described above for experiments.

Flow cytometry analysis

For analyses of purified CD8$^+$ T cells form peripheral blood, cells were gated by lymphocyte morphology and viability as shown in **Supp. Fig 1A.**

For analysis of cell surface protein expression, cells were stained in 1x PBS with 2% FCS (FACS buffer) with specific monoclonal antibodies and live/dead probe for 30 minutes at 4°C. Surface antibodies used are detailed in **Table S2**.

To assess proliferation, prior to the experiment, cells were incubated with cell trace violet (CTV, 5 µM Invitrogen, Cat# C34557) or carboxyfluorescein succinimidyl ester (CFSE, 5 µM, Invitrogen, Cat# C34554) for 20 mins at 37°C and 5% $CO_{2,}$ washed and cultured as described above, and analysed for CTV dilution at the end of the experiment.

For assessment of cytotoxic granule release and intracellular cytotoxic molecule or cytokine expression by intracellular staining, cells were first activated for 4 hours with Cell Activation Cocktail with Brefeldin A (PMA/Ionomycin, BioLegend Cat# 423303) or 12 µl/ml ImmunoCult Human CD3/CD28 T Cell Activator in combination with Brefeldin A (10µg/ml, Acros organics, Cat# 297140050) and monensin (Biolegend, Cat#420701) as indicated. Where degranulation was assessed, anti-CD107a (**Table S3**) was included during the culture period. Cells were then stained for viability as before, fixed using FoxP3 fixation/permeabilization

solution (eBioscience, Cat# 00-5523-00) for 20 mins at 4°C, washed with FoxP3 permeabilization buffer and incubated with antibodies for 30-60 minutes at 4°C. Antibodies used are detailed in **Table S3**.

To analyse phosphorylated proteins in the TCR signalling pathway, CD8$^+$ T cells were activated for the indicated time prior to fixation (Fixation Buffer, Biolegend, catalogue #420801, containing 4% paraformaldehyde), permeabilisation (True-Phos™ Perm Buffer, Biolegend, catalogue #425401) via dropwise addition of permeabilisation buffer and continuous vortexing, and staining for phospho-proteins (phopsho-Lck (P-Lck) and phospho-Erk (p-Erk) (**Table S3**) in FACS buffer.

For analysis of mTOR, p-p70S6K, c-Myc, BNIP3 and Rheb expression, as well as Akt, mTOR and p70S6K phosphorylation, cells were first fixed with pre-warmed FoxP3 fixation solution for 20 min at 37°C. Cells were then washed with FoxP3 permeabilization buffer and incubated with primary antibodies (**Table S3**) for 30 min at room temperature. After washing, cells were then incubated with secondary donkey anti-Rabbit antibody IgG1 (**Table S3**) for 20 min at room temperature if the primary antibody was not fluorophore-conjugated.

To assess mitochondrial mass, cells were incubated in RPMI/FCS with MitoView Green (50 nM; Biotium, Cat# 70054) for 20 mins at 37°C and 5% CO_2 prior to washing and analysis. To assess mitochondrial membrane potential ($\Delta\Psi m$) cells were incubated in RPMI/FCS with MitoSpy Orange (MSO, 25nM, Biolegend Cat# 424804) for 20 min at 37°C and 5% CO_2 ± the mitochondrial uncoupler, Bam-15 (3 µM, BioTechne, Cat# 5737) prior to washing and analysis. Data are presented as a ratio of MSO MFI in absence of Bam-15/ MSO MFI in presence of Bam-15.

To assess mitochondrial reactive oxygen species (mROS), cells were stained with mitoSOX (5µM, Invitrogen, Cat #M36008) in HBSS for 20 min at 37°C and 5% CO_2 prior to washing and analysis.

To assess total cellular reactive oxygen species (ROS) cells were stained with 2',7'-Dichlorofluorescin Diacetate (20µM DCFDA, Sigma Cat# D6883) in RPMI/FCS for 20 min at 37°C and 5% CO_2 prior to washing and analysis.

Nuclei isolation for NFAT analysis

Sucrose buffer A was prepared with 10 mM HEPES, 8 mM $MgCl_2$, 320 mM sucrose (Sigma-Aldrich, Cat #S0389), 0.1% Triton-X 100 (Sigma-Aldrich, Triton™ X-100, catalogue #X-100), protease and phosphatase inhibitor (Sigma-Aldrich, Protease and Phosphatase Inhibitor Cocktail, catalogue #PPC1010), and diluted with distilled H_2O. Sucrose buffer B was prepared as for 'sucrose buffer A' but without Triton-X 100. CD8$^+$ T cells were activated

with anti-CD3/anti-CD28 antibody cocktail (ImmunoCult™ Human CD3/CD28 T cell Activator, Stem Cell Technologies, catalogue #10971) for 1 hour in the indicated O_2 conditions prior to washing and incubation for 15 minutes on ice with 'sucrose buffer A'. $CD8^+$ T cells were centrifuged at 2000 g for 5 minutes at 4̊C before 2 washes with 'sucrose buffer B' at the same centrifuge settings. 16% paraformaldehyde (Thermo Scientific Chemicals, Paraformaldehyde 16% w/v aqn. Soln., methanol free, catalogue #043368-9M) was diluted with 'sucrose buffer B' to yield a 4% solution and used to fix the cells for 30 minutes on ice in the dark. After centrifugation and removal of the supernatant, the nuclei pellets were washed once with FACS buffer and $MgCl_2$ at 1000 g for 5 minutes at 4̊C, and once with Perm buffer and $MgCl_2$ at the same centrifuge settings. Nuclei were stained with fluorescent-labelled antibodies (**Table S3**) in Perm buffer and $MgCl_2$ for 1 hour at 4̊C. The nuclei pellets were washed twice in FACS buffer and $MgCl_2$ prior to running on the flow cytometer.

Western blotting analysis

Cell lysates were prepared in RIPA buffer (Thermo Fisher Scientific, Cat# 89900) and protein concentrations were determined with a BCA protein assay kit (for samples in RIPA only, Thermo Fisher Scientific, Cat# 23225). Whole-cell lysates were resolved by 10% SDS-PAGE and transferred onto nitrocellulose membranes. The membranes were then incubated with stained with primary and secondary antibodies (**Table S3**). The HRP-ECL system (Bio-Rad Clarity Western ECL Substrate Cat# 170-5061) was used for band detection.

Bispecific antibody-mediated cytotoxicity and T cell activation

BCMA-expressing multiple myeloma cell lines (JJN3, AMO and L363) were kindly provided by Professor Chris Bunce (University of Birmingham) and labelled with CTV prior to experiments (as above). $CD8^+$ T cells, isolated from healthy donor peripheral blood as above, were co-cultured with these cell lines at indicated T cell : target ratios ± BCMAxCD3 bispecific antibody (**Table S3**) at either 21% or 1% O_2 as indicated for 24 hours. Target cell death was quantified by flow cytometry analysis of live/dead probe exclusion within CTV-labelled target cells. In a parallel well, T cells and target cells were cultured as above, but additionally in presence of brefeldin A and monensin (details above). At the end of the assay, cells were surface stained for CD8, CD25 and CD69 prior to fixation and permeabilization as described above for intracellular cytokine and cytotoxic molecules, to permit quantification of abundance of these within CTV-negative CD8-positive T cells antibody details in **Table S3**.

ELISA

Cell culture supernatants were harvested at 24-72 hours as indicated and stored at -20°C before analysis by ELISA. IFN-γ concentration was measured using anti-IFN-γ capture (Thermo Fisher, Clone 2G1, Cat# M700) and biotinylated detection (Thermo Fisher, Clone B133.5, Cat# M701) antibodies, recombinant IFN-γ standard (Sigma-Aldrich Cat# SRP 3058), streptavidin-HRP (Sigma Aldrich, Cat# E2866) and TMB substrate (BD Biosciences, Cat# 555214). TNF-α concentration was measured using TNF alpha antibody pair and ELISA buffer kit (Invitrogen, Cat# CHC1753 and Cat# CNB0011).

Measurement of glucose and lactate in supernatants

Cell culture supernatants were harvested at 48 hrs and stored at -20°C before analysis. Glucose and lactate concentrations were measured using Glucose-Glo (Promega, Cat# J6021), Lactate-Glo (Promega, Cat# J5021) respectively, according to the manufacturer's instructions.

RNA-sequencing and analysis

Cultured cells were harvested, and mRNA was isolated using NucleoSpin RNA Mini Kit (Machery-Nagel, Cat# 740955.5) according to the manufacturer's instructions. RNA samples were submitted to Genomics Birmingham for RNA Sequencing. Sequencing libraries were prepared using the QuantSeq 3' mRNA-Seq Library Preparation Kit (Lexogen). Libraries were sequenced using the NextSeq 500 with a Mid 150v2.5 flow cell. FASTQ files were downloaded from Illumina base space and uploaded to BlueBEAR (University of Birmingham) for further analysis. All 4 lanes were merged to create the final FASTQ file for each sample which were then quality checked by FASTQC. Bbduk v37.99 from the bbmap suite was used for trimming low-quality read, poly(A) tails, rRNA and adapter contamination. STAR v2.7.2b aligner was used to align reads to the human genome. HTSeq-count v0.13.5 was used to generate counts for mRNA species, raw read counts were then used for further analysis by DESeq2 v1.30.1(19) in R v4.0.5. Data were filtered to remove genes where 6 or fewer samples had a read count of 10 or above. Differentially expressed genes (DEGs) were selected as those with an adjusted p value < 0.05. For individual genes, the normalised counts are presented. Heatmap analysis was performed on the normalised count data using the R package gplots. For gene ontology pathway analysis, org.Hs.eg.db v3.12.0(20) and clusterProfiler v3.18.1(21) packages were used. Venn diagram generated using Venny v2.1(22).

Stable isotope based metabolic tracing

Cells (4 x 10^6 per condition) were cultured as above for 24 hours in SILAC RPMI 1640 (Gibco, Cat# A2494201) supplemented with 10% FCS, rhIL-2, 20 mg/ml L-arginine, 4 mg/ml L-lysine (both Sigma-Aldrich), and either 2 mM U-$^{13}C_5$ glutamine (CK Isotopes, Cat# CNLM-1275) and 10 mM glucose (Sigma Aldrich) or 2mM L-glutamine (Sigma Aldrich) and 10 mM U-$^{13}C_6$ glucose (CK Isotopes, Cat# CLM-1396-1). Cells were then washed with ice-cold 0.9% saline solution and were extracted in 5:2:5 pre-chilled HPLC-grade methanol, HPLC-grade water (containing 1.75 µg/mL D6-glutaric acid) and chloroform. The extracts were shaken at 1400 rpm for 15 min at 4°C and centrifuged at 12,000 g for 15 min at 4°C. The upper aqueous phase was collected and evaporated under vacuum. Metabolite derivatization was performed using a two-stage derivatization protocol. Dried polar metabolites were dissolved in 20 µL of 2% methoxyamine hydrochloride in pyridine (Thermo Fisher Scientific, Cat# 25104) at 60°C for 60 minutes, followed by 30µl of N-tert-Butyldimethylsilyl-N-methyltrifluoroacetamide with 1% tertbutyldimethylchlorosilane, incubated at 60°C for 60 minutes. GC-MS analysis was performed using an Agilent 6890GC equipped with a 30m Rxi-5ms (0.25 mm ID) capillary column. The GC was connected to an Agilent 5975C MS operating under electron impact ionization at 70 eV. The MS source was held at 230°C and the quadrupole at 150°C. The detector was operated in scan mode and 1 µL of derivatised sample was injected in splitless mode. Helium was used as a carrier gas at a flow rate of 1 mL/min. The GC oven temperature was held at 100°C for 1 min and increased to 160°C at a rate of 10°C/min, increased to 200°C at 5°C/min, and a final increase to 330°C at 10°/min and held for 4 minutes. The run time for each sample was 32 min. For determination of the mass isotopomer distributions (MIDs), spectra were corrected for natural isotope abundance. Data processing was performed using MATLAB.

CRISPR-Cas9 editing of BNIP3 expression

Isolated $CD8^+$ T cells were rested in 21% O2 for 48 hours in RPMI-1640 containing 100 IU/ml penicillin and streptomycin, 10% FCS, and 50 IU/ml recombinant IL-2, additionally supplemented with 50 ng/mL IL-7 (Biolegend, Cat #581904) and 50 ng/mL IL-15 (Biolegend, Cat# 570302). BNIP3 and control crRNA (Integrated DNA Technologies, KO:Hs.Cas9.BNIP3.1.AA, Alt-R® Clustered Regularly Interspaced Short Palindromic Repeats (CRISPR)-CRISPR associated protein 9 (Cas9) crRNA; Negative control: Alt-R® Cas9 Negative Control crRNA, Cat# 1072544) and tracRNA were resuspended in nuclease-free duplex buffer (Integrated DNA Technologies, Cat# 11-01-03-01) at 200 µM. Duplex RNA was generated by mixing 1:1 BNIP3 or control crRNA with tracRNA in a sterile Eppendorf and boiling at 95°C for 5 minutes. Duplex RNA was cooled at room temperature until use.

RNP complexes were generated by mixing duplex RNA with Alt-R™ S.p. HiFi Cas9-green fluorescent protein (GFP) V3 (Integrated DNA Technologies, Cat# 10008100) in a 3:1 ratio. For RNP complex formation, these were incubated for 15 minutes at room temperature and then kept on ice until use. $CD8^+$ T cells were counted, and 2 x 10^6 cells resuspended in 40-100 ul P3 buffer with supplement added (P3 Primary Cell Solution box, Lonza, Cat# PBP3-00675). 5.86 µL of RNP complex was added to each sample. Cells were transfected in a NucleofectorTM Transfection II/2b device (Lonza) with the 'Unstimulated T cells, High Efficiency' program code. Transfected cells were resuspended in pre-warmed RPMI-1640 containing 100 IU/ml penicillin and streptomycin, 20% FCS, and 50 IU/ml recombinant IL-2 and transferred to a 24 well plate for resting in 21% O_2, 37°C and 5% CO_2 overnight. The following day, $CD8^+$ T cells were resuspended in RPMI-1640 containing 100 IU/ml penicillin and streptomycin, 10% FCS, and 50 IU/ml recombinant IL-2 at a density of 1 x 10^6/ml. Cells were moved to 21% or 1% O_2 and stimulated as described above 2 hours later. Cells were cultured for 72 hours in 21% or 1% O2. Following culture, transfected $CD8^+$ T cells were stained for viability, surface markers and intracellular antibodies and cytokines detailed above.

Flow cytometry analysis of multiple myeloma patient samples

Mononuclear cells were isolated by density-gradient centrifugation using Ficoll-Paque (Cytiva, Cat# 17144003), harvested, washed with phosphate-buffered saline (PBS, Sigma, Cat# D8537), counted and frozen in freezing medium (10% DMSO, Sigma, Cat# 472301, 90% Foetal Calf Serum (FCS), Sigma, Cat# F9665). Cells were then thawed, washed twice in RPMI (Sigma, Cat# R8758), resuspended at 1x10^6/ml in RPMI/FCS then rested at 37°C/5%CO_2 for 16 hours. Cells were stained for viability with efluor780 (Invitrogen, Cat# 13539140) and fluorophore-conjugated antibodies for cell surface markers (**Table S3**) as above. Cells were washed twice with FACS buffer and analysed using a BD LSRFortessa X-20 flow cytometer. For intracellular cytokine staining, T cells were stimulated using Immunocult CD3/28 T cell activator (StemCell, Cat# 10971) and Brefeldin A (10µg/ml, Acros organics, Cat# 297140050) for 4 hours, stained for viability and cell surface markers before fixation/permeabilization (FoxP3 buffer set, ebioscience Cat# 00-5523-00) and staining with anti-cytokine fluorophore-conjugated antibodies (**Table S3**). For Ki67, c-Myc and Rheb staining, cells were fixed and permeabilised and stained as described above. The gating strategy for identification of total $CD8^+$ T cells within these samples is shown in **Supp. Fig 6A** and is based on morphology, viability, expression of CD8 and lack of

expression of CD4 and CD56. $CD8^+$ T cells were further gated on naïve, central memory, effector memory and EMRA populations as indicated in **Fig. 6B.**

Statistical analysis

Data are presented as the mean and individual replicate values. Paired data were analysed by paired Student's *t* test for two conditions, repeated measures ANOVA with Holm-Sidak's post-hoc test for more than two conditions or two-way ANOVA with Holm-Sidak's post-hoc test for more than one parameter. Unpaired data were analysed by unpaired *t* test. Specific tests used are indicated in the figure legend. Analysis was performed using GraphPad Prism 9.

.

Table S1: General and Clinical Characteristics of Patients

n	8
Age – Median (Range)	74 (67-86)
Sex - % male	63
Trephine % MMPC - Median (Range)	60 (45-80)
PP level (g/L) - Median (Range)	41.7 (16.8-74.2)
IgG (%)	37.5
IgA (%)	50
Light Chain (%)	12.5

Table S2: Breakdown of relevant clinical characteristics for each patient

Study ID	Sex	Age	Trephine % MMPC	PP level (g/L)	PP type	Beta-2 micro-globulin (mg/L)	Albumin (g/L)	ISS	Cytogenetics
QE147	M	72	50	57	IgG κ	7.8	32	3	ND
QE158	M	74	45	16.8	IgA κ	3	38	1	ND
QE163	F	86	60	74.2	IgG κ	22.8	23	3	ND
QE190	M	72	60	33.7	IgA κ	2.9	34	2	ND
QE206	M	81	50	None	κ Light Chain	4.2	42	2	ND
QE233	F	67	70	41.7	IgG κ	2.9	30	2	+1q; + 2 copies FGFR3, CCND1, CH17, MAFB; +1 copy IGH; -1 copy MAF
QE263	F	79	75	34.5	IgA λ	3.6	36	2	ND
QE344	M	74	80	55.1	IgA κ	8.6	29	3	+1q; IGH rearranged (unknown partner)

ND = Not Done; ISS = International Staging System

All patients were newly diagnosed and had not received previous treatment for multiple myeloma

Table S3: Antibody and reagent details

Antibody	Clone	Fluorophore	Supplier	Cat#	Conc.
eFluor™ 780 Viability Dye	N/A	APC-Cy7	Invitrogen	65-0865-14	1 in 1000
Zombie Violet Viability Dye	N/A	BV421	Biolegend	423114	1 in 500
Anti-human CD25	BC96	BV605	Biolegend	302632	1 in 50
Anti-human CD69	FN50	APC	Biolegend	310910	1 in 50
Anti-human CD8	SK1	AF700	Biolegend	344724	1 in 50
Anti-human CD4	OKT4	BV785	Biolegend	317442	1 in 50
Anti-human CD56	HCD56	BV510	Biolegend	318340	1 in 50
Anti-human CD62L	DREG-56	AF647	Biolegend	304818	1 in 50
Anti-human CD45RA	HI100	BV421<br>BV605	Biolegend	304130<br>304135	1 in 50
Anti-human Ki67	11F6	FITC	Biolegend	151212	1 in 50
Anti-human IFN-gamma	B27	FITC	Biolegend	506504	1 in 50
Anti-human TNF-alpha	MAb11	PE	Biolegend	502909	1 in 150
Anti-human Granzyme B	GB11	AF700	BD	561016	1 in 50
Anti-human CD107a	H4A3	FITC	BD	555800	1 in 50
Anti-human Perforin A	Delta G9	PE-Cy7	Invitrogen	25-9994-42	1 in 50
Anti-human/mouse Phospho-mTOR (Ser2448)	MRRBY	PE	Invitrogen	12-9718-42	1 in 100
Donkey anti-rabbit IgG	Polyclonal	AF555	Invitrogen	A31572	1 in 500
Donkey anti-rabbit IgG	Polyclonal	BV421	Biolegend	406410	1 in 100
Anti-human/mouse ERK1/2 Phospho (Thr202/Tyr204)	4B11B69	AF647	Biolegend	675504	1 in 50
Anti-human/mouse Lck Phospho (Tyr505)	A17013A	PE	Biolegend	699704	1 in 50
Anti-human/mouse NFAT1 XP(R) Rabbit mAb	D43B1	AF647	Cell Signalling	14201S	1 in 50
Anti-human/mouse P-p70 S6 Kinase (T421/S424) Rabbit Ab	Polyclonal	Unconjugated	Cell Signalling	9204S	1 in 100
Anti-human/mouse P-Akt (T308) Rabbit mAb	C31E5E	Unconjugated	Cell Signalling	2965L	1 in 100
Anti-human c-Myc Rabbit mAb	E5Q6W	Unconjugated	Cell Signalling	18583S	1 in 200
Anti-human BNIP3 Rabbit mAb	D7U1T	Unconjugated	Cell Signalling	44060S	1 in 100
Anti-human Rheb mAb	GT39819	Unconjugated	Invitrogen	MA5-27777	1 in 100
BCMAxCD3 Bispecific Ab	N/A	Unconjugated	Invivogen	bimab-hbcmacd3-05	10 ng/ml

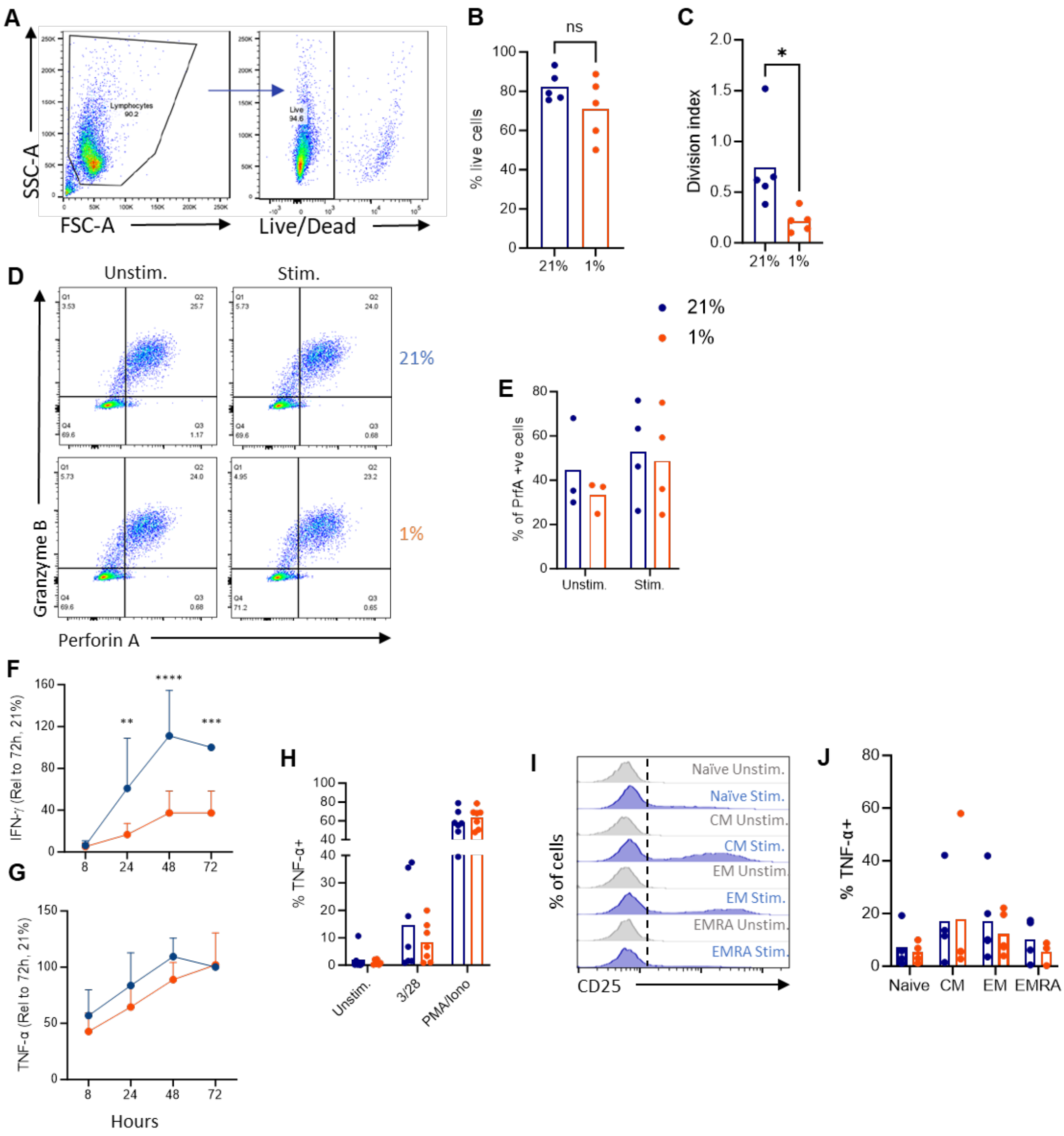

Supplementary Figure 1 (Related to Figure 1)

(**A**) Example gating strategy for flow cytometry analyses of purified CD8$^+$ T cell cultures, permitting analysis of viable CD8$^+$ T cells. (**B-C**) CD8$^+$ T cells were cultured for 16 hours at 21% or 1% O_2, stimulated via CD3/CD28 for 6 days at these oxygen tensions and assessed for (**A**) viability and (**B**) proliferation by cell trace violet (CTV) dilution, measured by flow cytometry (summarised for n=5 independent donors, division index is calculated from CTV fluorescence as the average number of cell divisions a cell in the original population has undergone, which includes the undivided peak. (**C-D**) CD8$^+$ T cells were cultured as in (A) but stimulated for 5 hours in presence of brefeldin A and monensin, and assessed for intracellular abundance of granzyme B (GzmB) and Perforin A (PrfA) by flow cytometry (C, representative plots and (D) summarised data for PrfA for n= independent donors). (**E-F**) Supernatants from CD8$^+$ T cells cultured as in (A) for indicated duration were assessed for (**E**) IFN-γ and (**F**) TNF-α content by ELISA (summarised data for n=6 independent donors, normalised to total cytokine measured at 72 hours at 21% O_2). (**G**) CD8$^+$ T cells cultured at indicated % O_2 as in (A) were restimulated at 48 hours via CD3/CD28 or with PMA/ionomycin as indicated and assessed for intracellular TNF-α abundance by flow cytometry (summarised for n=7 independent donors). (**H-I**) Flow-cytometry sorted populations of naïve (CD45RA$^+$CD62L$^+$), central memory (CM, CD45RA$^-$CD62L$^+$), effector memory (EM, CD45RA$^-$CD62L$^-$) and EMRA (CD45RA$^+$CD62L$^-$) cells were cultured at (**H**) 21% O_2 ± CD3/CD28 stimulation and assessed for surface CD25 expression by flow cytometry, or (**I**) 21% O_2 or 1% O_2 and assessed for intracellular TNF-α abundance by intracellular staining and flow cytometry (summarised for n=3-5 independent donors). p values were calculated by (B) paired t test and (E) two-way ANOVA and Holm-Sidak's post-hoc test. * $p < 0.05$, ** $p < 0.01$, *** $p < 0.001$, **** $p < 0.0001$.

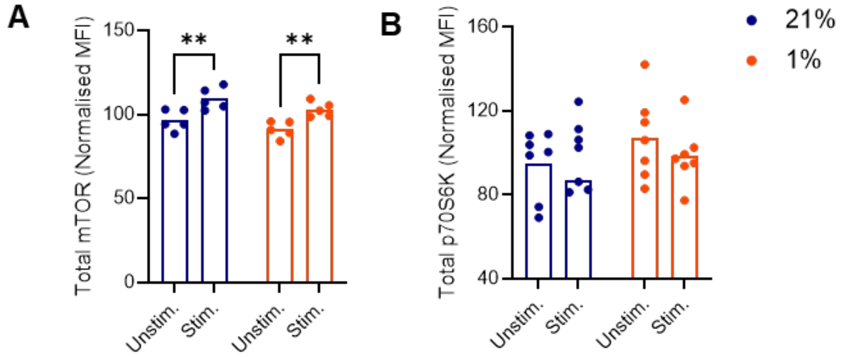

Supplementary Figure 2 (Related to Figure 2)

CD8[+] T cells were cultured for 16 hours at 21% or 1% O_2, stimulated via CD3/CD28 as indicated at these oxygen tensions as assessed after 24 hours for intracellular abundance of (**A**) total mTOR and (**B**) total p70S6K by flow cytometry (summarised data for n=5-7 independent donors, shown either as raw mean fluorescence intensity (MFI) or normalised to the average (mean) MFI for all matched samples from each donor). p values were calculated by two-way ANOVA and Holm-Sidak's post-hoc test. * $p < 0.05$, ** $p < 0.01$.

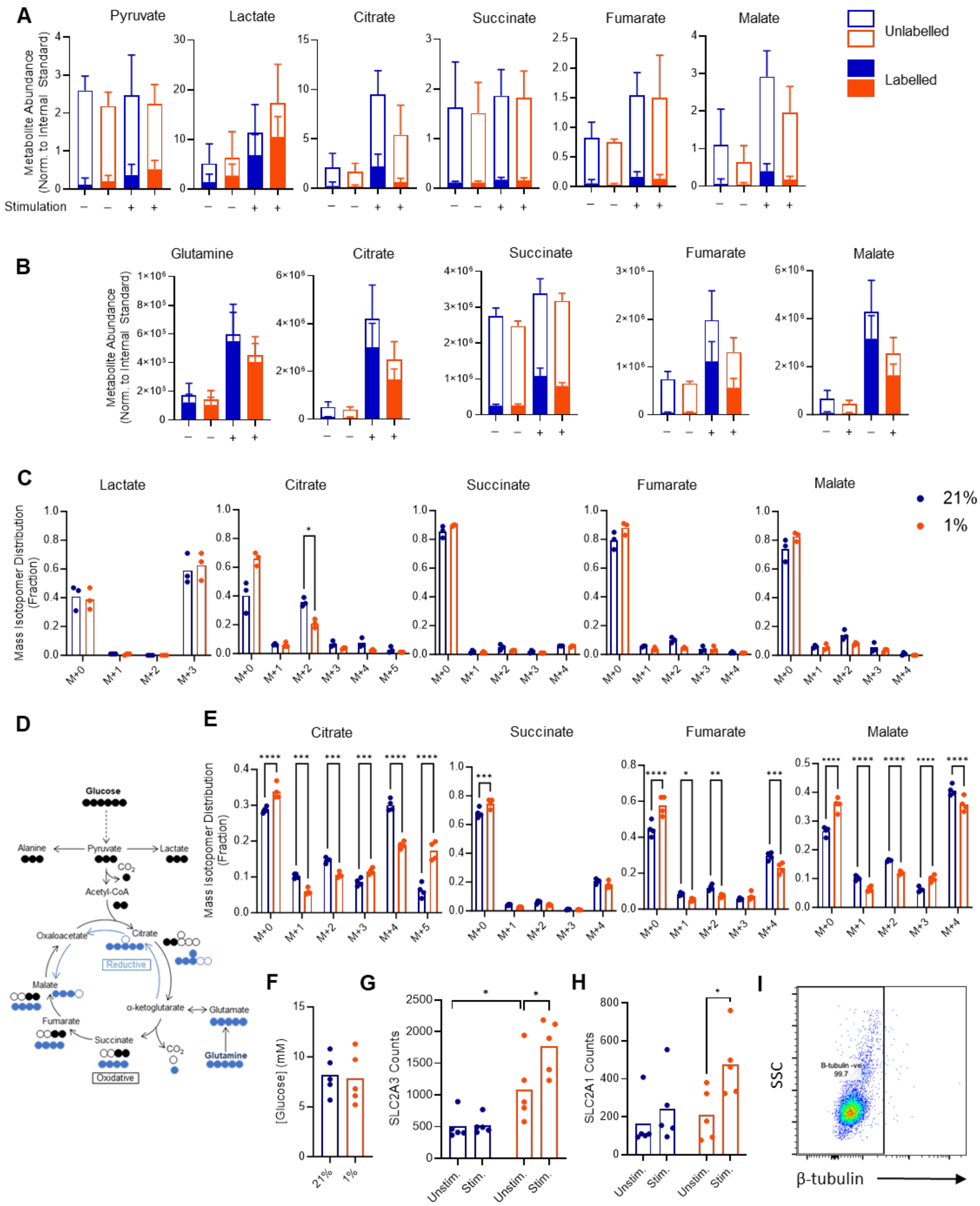

Supplementary Figure 3 (Related to Figure 3)

(**A-E**) CD8+ T cells were cultured for 16 hours at 21% or 1% O_2, stimulated via CD3/CD28 at these oxygen tensions as indicated for 24 hours in presence of fully-labelled ^{13}C-glucose or ^{13}C-glutamine and assessed for (**A-B**) total and labeled abundance and (**C,E**) mass isotopomer distribution of indicated metabolites by GC-MS (n=4 independent donors). (**D**) Schematic indicating mass isotopomers arising from ^{13}C-glucose and ^{13}-glutamine, through oxidative and reductive TCA activity (**F**) CD8+ T cells were cultured for 16 hours at 21% or 1% O_2, stimulated via CD3/CD28 at these oxygen tensions as indicated for 48 hours then assessed for glucose consumption by measurement of residual supernatant concentration (n=5 independent donors). (**G-H**) mRNA counts for Glut3 (SLC2A3) and Glut1 (SLC2A1) in cells cultured as in (A-D), analysed by RNA-sequencing (n=5 independent donors). (**I**) CD8+ T cells cultured as in (B) for 24 hours were fractionated by sucrose gradient centrifugation to isolate nuclei and assessed for β-tubulin presence to confirm nuclei isolation and permit gating on successfully isolated nuclei (β-tubulin-negative population, representative flow cytometry plot). p values assessed by (, E,G-H-) two-way ANOVA and Holm-Sidak's post-hoc test. * $p < 0.05$, ** $p < 0.01$, *** $p < 0.001$, **** $p < 0.0001$.

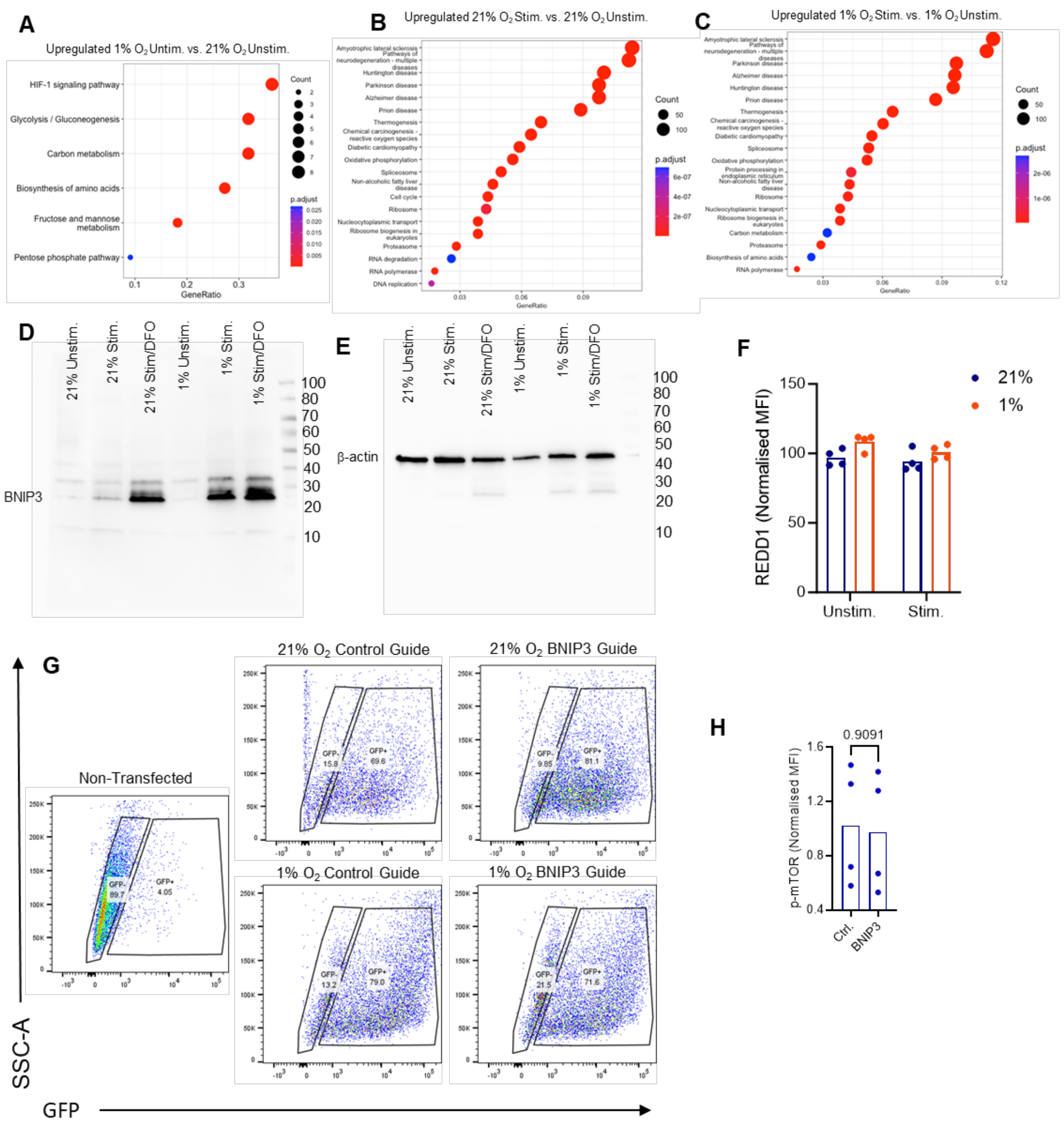

Supplementary Figure 4 (Related to Figure 4)

CD8$^+$ T cells were cultured for 16 hours at 21% or 1% O_2, then left unstimulated or stimulated via CD3/CD28 at these oxygen tensions for 24 hours prior to RNA-sequencing analysis (n = 5 independent donors). (**A-C**) Pathway analysis of genes upregulated in (**A**) unstimulated cells cultured at 1% O_2 compared to 21% O_2, (**B**) cells stimulated at 21% O_2 compared to matched unstimulated cells and (**C**) cells stimulated at 1% O_2 compared to matched unstimulated cells. (**D-E**) Example western blot for (**D**) BNIP3 and (**E**) beta-actin on CD8+ T cells activated as in (A-C). (**F**) Summary of intracellular flow cytometry analysis of REDD1 expression in CD8$^+$ T cells stimulated as above for 24 hours (n = 4 independent donors). (**G-H**) CD8$^+$ T cells were transfected with control (Ctrl.) or BNIP3-targeting CRISPR guide RNAs and green fluorescent protein (GFP)-tagged Cas9 and stimulated at 21% or 1% O_2 for 24 hours. (**G**) Example plots showing GFP+ cell frequencies in indicated conditions and gating strategy for analysis of GFP+ cells (**H**) phospho-mTOR MFI within indicated GFP+ cells cultured at 21% O_2, analysed by flow cytometry (summarised data for n=4 independent donors, normalised to the average (mean) MFI for the two matched samples from each donor).

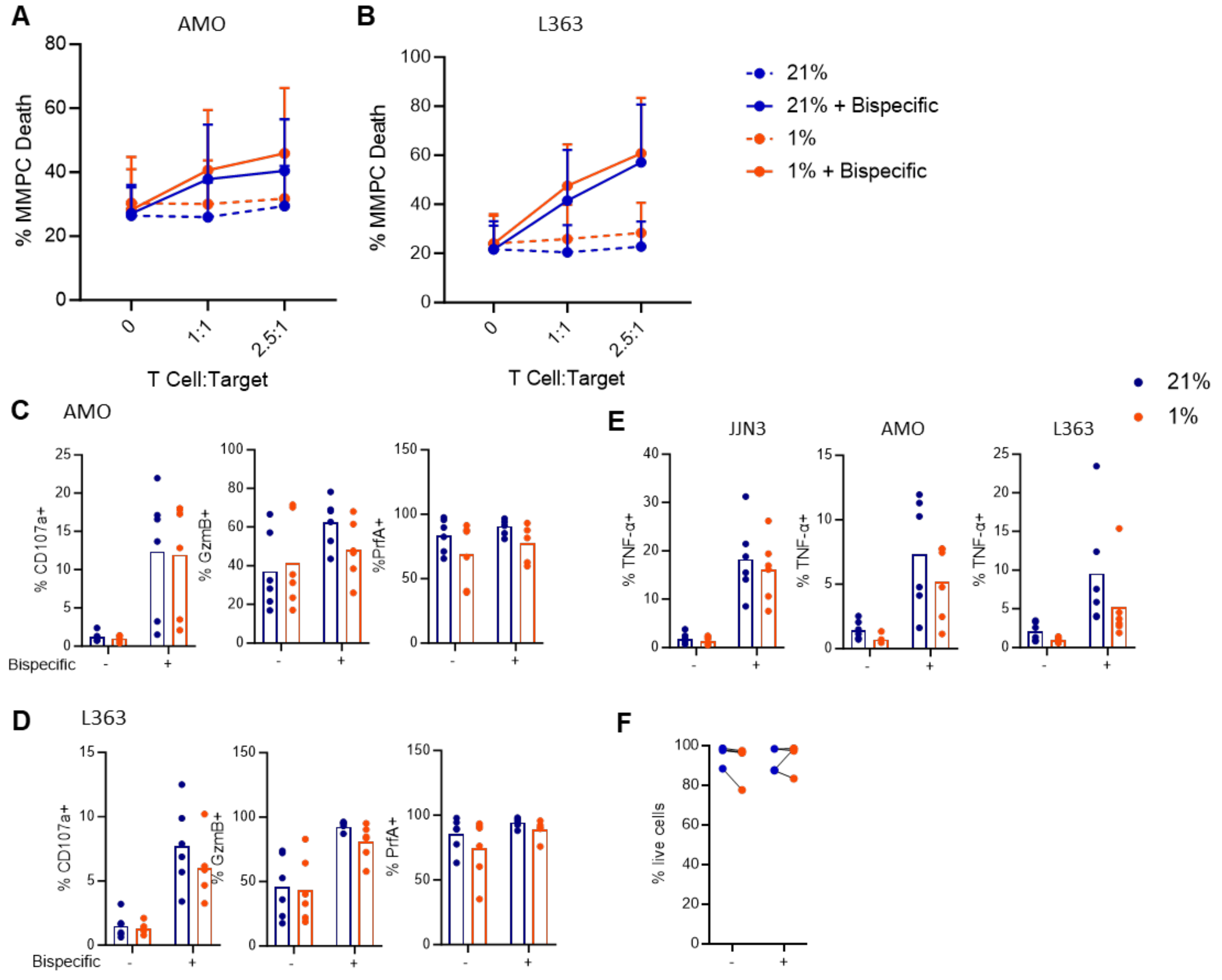

Supplementary Figure 5 (Related to Figure 5)

(**A-B**) CD8⁺ T cells were cultured with BCMA-expressing, CTV-labeled (**A**) AMO and (**B**) L363 target cells and BCMAxCD3 bispecific antibody where indicated for 24 hours at either 21% O_2 or 1% O_2, prior to assessment of target cell viability (live/dead probe exclusion) by flow cytometry (summarised data for n=5 independent donors of CD8⁺ T cells across indicated T cell : target ratios). (**C-E**) CD8⁺ T cells and (**C**) AMO (**D**) L363 or (**E**) indicated target cells were cultured as above, additionally in presence of brefeldin A/monensin and CD8⁺ T cells assessed for (**C-D**) CD107a trafficking, intracellular granzyme B (GzmB) and perforin A (PrfA) or (**E**) intracellular TNF-α by flow cytometry (summarised data for n=5 independent donors of CD8⁺ T cells at 2.5 T cells : 1 target cell ratio). p values were calculated by two-way ANOVA and Holm-Sidak's post-hoc test. * $p < 0.05$, ** $p < 0.01$, *** $p < 0.001$, **** $p < 0.0001$.

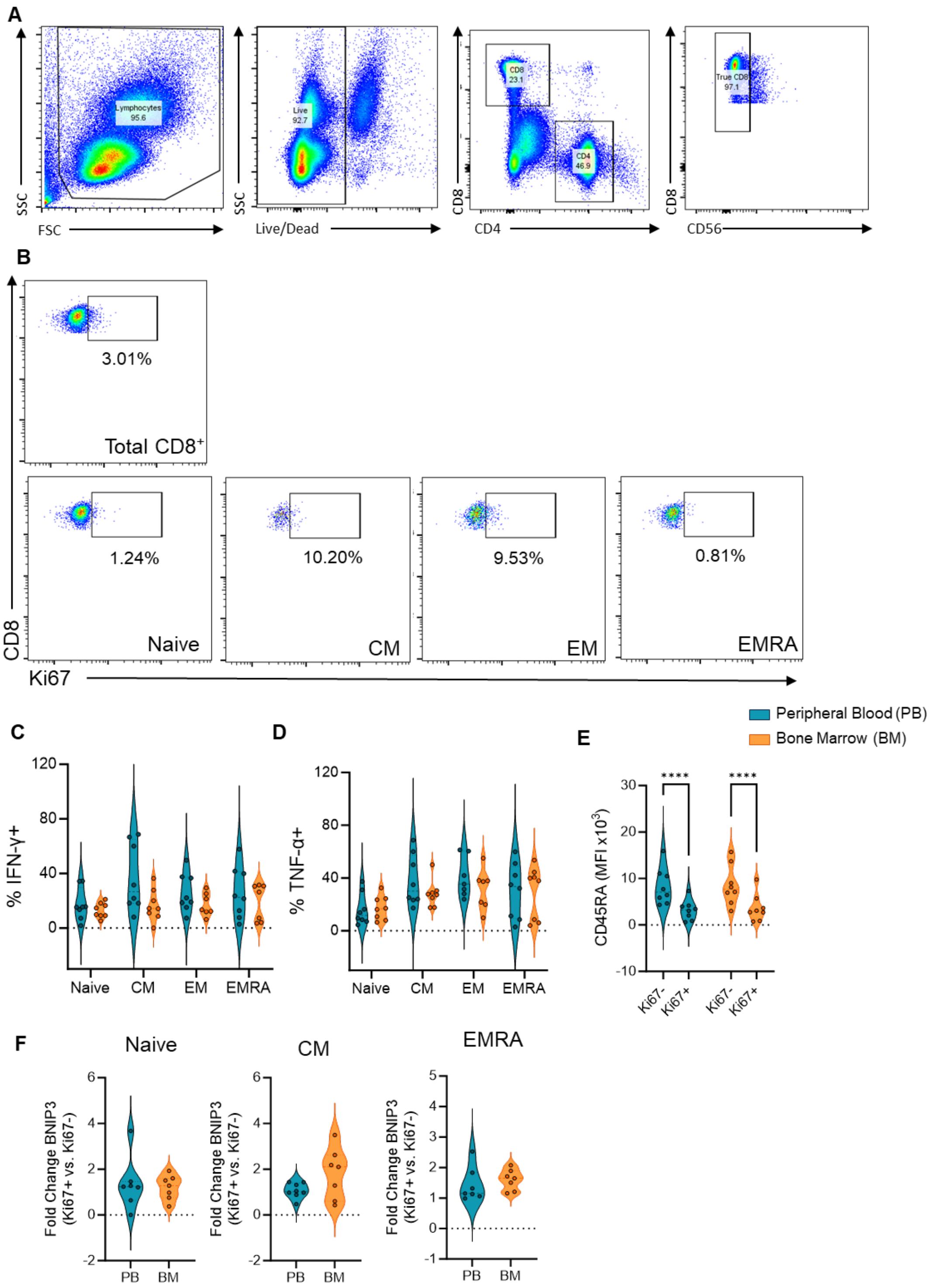

Supplementary Figure 6 (Related to Figure 6)

(**A**) Gating strategy for analysis of CD8$^+$ T cells within mononuclear cells from bone marrow (BM) and peripheral blood (PB) of patients with newly-diagnosed multiple myeloma. (**B**) Flow cytometry analysis of Ki67 expression within naïve, CM, EM and EMRA CD8$^+$ T cells of these samples (representative flow cytometry plots for total CD8+ T cells and indicated populations). (**C-D**) Paired bone marrow (BM) and peripheral blood (PB) mononuclear cells from patients with newly-diagnosed multiple myeloma were stimulated for 4 hours with anti-CD3/28 and analysed by flow cytometry for (**C**) IFN-γ and (**D**) TNF-α expression within indicated populations. (**E-F**) Samples as in (C-D) were analysed directly ex vivo for frequency of Ki67-expressing cells within (**E**) CD45RA+ and CD45RA- cells as indicated and (**F**) fold change of BNIP3 abundance in Ki67$^+$ vs. Ki67$^-$ cells of indicated populations. For all analyses n=8 independent paired samples. p values were calculated by two-way ANOVA and Holm-Sidak's post-hoc test. **** $p < 0.0001$.
